# Cytokinin stabilizes WUSCHEL by acting on the protein domains required for nuclear enrichment and transcription

**DOI:** 10.1371/journal.pgen.1007351

**Published:** 2018-04-16

**Authors:** Stephen A. Snipes, Kevin Rodriguez, Aaron E. DeVries, Kaori N. Miyawaki, Mariano Perales, Mingtang Xie, G. Venugopala Reddy

**Affiliations:** Department of Botany and Plant Sciences, Center for Plant Cell Biology (CEPCEB), Institute of Integrative Genome Biology (IIGB), University of California, Riverside, California, United States of America; Nara Institute of Science and Technology, JAPAN

## Abstract

Concentration-dependent transcriptional regulation and the spatial regulation of transcription factor levels are poorly studied in plant development. WUSCHEL, a stem cell-promoting homeodomain transcription factor, accumulates at a higher level in the rib meristem than in the overlying central zone, which harbors stem cells in the shoot apical meristems of *Arabidopsis thaliana*. The differential accumulation of WUSCHEL in adjacent cells is critical for the spatial regulation and levels of *CLAVATA3*, a negative regulator of *WUSCHEL* transcription. Earlier studies have revealed that DNA-dependent dimerization, subcellular partitioning and protein destabilization control WUSCHEL protein levels and spatial accumulation. Moreover, the destabilization of WUSCHEL may also depend on the protein concentration. However, the roles of extrinsic spatial cues in maintaining differential accumulation of WUS are not understood. Through transient manipulation of hormone levels, hormone response patterns and analysis of the receptor mutants, we show that cytokinin signaling in the rib meristem acts through the transcriptional regulatory domains, the acidic domain and the WUSCHEL-box, to stabilize the WUS protein. Furthermore, we show that the same WUSCHEL-box functions as a degron sequence in cytokinin deficient regions in the central zone, leading to the destabilization of WUSCHEL. The coupled functions of the WUSCHEL-box in nuclear retention as described earlier, together with cytokinin sensing, reinforce higher nuclear accumulation of WUSCHEL in the rib meristem. In contrast a sub-threshold level may expose the WUSCHEL-box to destabilizing signals in the central zone. Thus, the cytokinin signaling acts as an asymmetric spatial cue in stabilizing the WUSCHEL protein to lead to its differential accumulation in neighboring cells, which is critical for concentration-dependent spatial regulation of *CLAVATA3* transcription and meristem maintenance. Furthermore, our work shows that cytokinin response is regulated independently of the WUSCHEL function which may provide robustness to the regulation of WUSCHEL concentration.

## Author summary

Stem cell regulation is critical for the development of all organisms, and plants have particularly unique stem cell populations that are maintained throughout their lifespan at the tips of both the shoots and roots. Proper spatial and temporal regulation of gene expression by mobile proteins is essential for maintaining these stem cell populations. Here we show that in the shoot, the mobile stem cell promoting factor WUSCHEL is stabilized at the protein level by the plant hormone cytokinin. This stabilization occurs in a tightly restricted spatial context, and movement of WUSCHEL outside of this region results in WUSCHEL instability that leads to its degradation. The specific regions on the WUSCHEL protein that respond to the cytokinin signaling are the same regions that are essential for both proper WUSCHEL localization in the nucleus and regulation of its target genes. This spatially specific response to cytokinin results in differential accumulation of WUSCHEL in space, and reveals an intrinsic link between protein stability and the regulation of target genes to maintain a stable population of stem cells.

## Introduction

Plant meristem development depends largely on positional information. Determining how cells interpret positional cues to regulate gene expression is central to cell fate specification during pattern formation. The concentration of transcription factors (TFs) in developmental fields have been shown to provide positional information in gene expression and cell fate specification in animal systems [1, 2, 3]. The concentration-dependent transcriptional regulation is not well-studied in plants. Recently, the homeodomain TF-WUSCHEL (WUS) has been shown to regulate stem cell gene expression in a concentration-dependent manner. Stem cells located in the central zone (CZ) of the shoot apical meristems (SAMs) of plants sustain the growth and development of all aboveground plant parts [4]. A subset of stem cell daughters that divide relatively infrequently remains in the CZ [5, 6, 7]. The other stem cell daughters that are displaced into the adjacent peripheral zone (PZ) divide rapidly and differentiate as lateral organs, while those that are displaced basally into the rib meristem (RM) differentiate as pith cells and become part of the stem [4]. *WUS* expressed in the RM is essential for the maintenance of stem cells in the overlying CZ [8, 9, 10]. WUS protein has been shown to migrate into adjacent cells [11], likely through plasmodesmata [12] where it accumulates at a lower level than in the RM. WUS promotes stem cell fate in the CZ, regulates cell division patterns, and also activates *CLV3* [9, 13, 14]. CLV3 is a secreted peptide that activates a receptor kinase pathway to restrict *WUS* transcription [15–18]. A recent study has shown that WUS binds the same cis-elements to activate and repress *CLV3* at lower and higher levels, respectively [13]. The concentration-dependent transcriptional regulation has been shown to control *CLV3* levels and spatial accumulation. Thus the differential accumulation of WUS in the RM and in the adjacent CZ is critical for regulating *CLV3*, which in turn impacts *WUS* transcript levels and the overall size of the SAM [19].

Rodriguez et al. [20] have shown that several interdependent processes involving DNA dependent homodimerization, nuclear-cytoplasmic partitioning, and protein destabilization contribute to higher WUS accumulation in the RM than in the adjacent cells. A specific amino acid residue in the DNA binding homeodomain (referred to as HOD1) is required for both DNA binding and homodimerization. However, HOD1 is not sufficient for full homodimerization, as a second part of the protein (aa134-aa208) also mediates homodimerization (referred to as HOD2). The mHOD1 (single amino acid missense mutation G77E) and ΔHOD2 (deletion of amino acids 134–208) double mutants of WUS have been shown to have higher diffusivity, which implicates DNA binding and homodimerization in the spatial accumulation of WUS. Also, the last 63 amino acid stretch of WUS that contains the acidic region, the WUS-box and the EAR-like domain has been shown to be sufficient for differential accumulation of WUS in the RM and the CZ [20]. A combined analysis of the point mutations in single and double mutants of the WUS-box and the EAR-like domain suggested that the EAR-like domain functions as a nuclear export signal, while the WUS-box functions as a nuclear retention signal, which implicates the nuclear-cytoplasmic partitioning dynamics in the regulation of nuclear concentration [20]. Moreover, ectopic overexpression of the wild type WUS in the CZ resulted in lower WUS protein accumulation [13]. On the contrary, the ectopic overexpression of WUS carrying a potent nuclear localization signal accumulated stably in cells of the CZ, showing that nuclear enrichment leads to WUS protein stability [13]. These studies suggest that higher WUS levels leads to its destabilization, until it reaches a saturating concentration where it becomes stable. Furthermore, a conditional nuclear translocation of WUS using the Dexamethasone (Dex) inducible form of WUS led to low or undetectable amount of the protein within 24 hours of Dex treatment in the CZ cells [20]. In contrast, the cells in deeper cell layers of the RM and the PZ accumulated WUS protein, suggesting that these cells may contain signals that can protect WUS from destabilizing signal/s. Therefore, positional signals and mechanisms that regulate differential accumulation of WUS in SAMs requires further investigation.

The higher WUS accumulation in the RM could simply be due to the synthesis of the protein in these cells [9, 11]. Earlier studies have shown that the exogenous application of cytokinin can induce *WUS* promoter activity in the RM [21, 22]. Recent studies have shown that the type-B ARRs that function downstream of the cytokinin receptor system bind the *WUS* promoter and activate transcription [23]. The synthetic cytokinin sensing promoter-*pTCSn* is active in cells of the RM while its activity is excluded from the L1 and the L2 layers [24, 25], which is explained based on the localized expression of cytokinin receptors in the RM [26]. The higher cytokinin signaling could be established by the WUS-mediated repression of type A *ARABIDOPSIS RESPONSE REGULATORS* (*ARRs*), negative regulators of cytokinin signaling [27]. Therefore, synthesis of WUS in the RM, promoted by the cytokinin signaling, may lead to higher WUS protein accumulation. However, as shown by the ectopic overexpression experiments explained in the earlier section, the higher synthesis alone would not lead to a uniformly higher protein accumulation. This suggests that additional levels of post-transcriptional regulation mediated by the positional signals could be important.

Here we present evidence that the RM-localized cytokinin signaling is necessary for promoting stability of the WUS protein, and acts on the acidic domain and the WUS-box transcriptional regulatory domains to stabilize the protein. We show that the Dex inducible form of the transcriptionally inactive, WUS-box mutant form of WUS fails to destabilize itself despite nuclear translocation, providing evidence that the WUS-box functions as a degron sequence. We also demonstrate that cytokinin can negate destabilization of WUS. The work presented here enriches the prevailing paradigm by providing a framework which involves a sensitive role for cytokinin signaling in promoting WUS protein stability in addition to its expression. The multifaceted WUS-box required for nuclear retention, cytokinin sensing, degradation and transcription acts as a common link that differentially interprets spatial cues to regulate the differential WUS accumulation required for concentration-dependent regulation of *CLV3* transcription.

## Results

### Exogenous application of cytokinin leads to higher WUS protein accumulation

**Fig 1:**
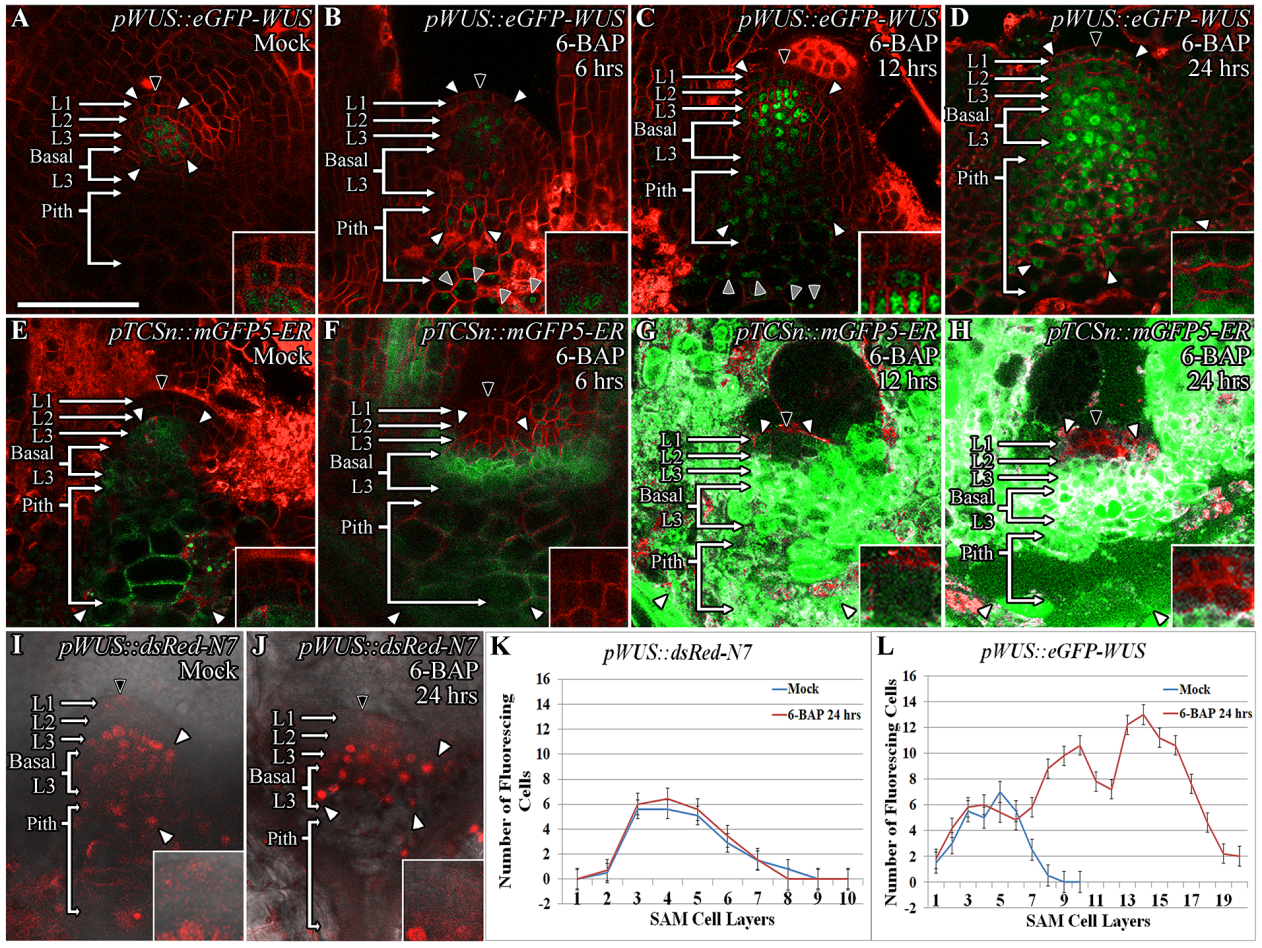
Exogenous application of cytokinin leads to higher WUS protein accumulation which corresponds with the spatial pattern of induction of cytokinin response. Side views of SAMs showing WUS protein **(***pWUS*::*eGFP-WUS*) accumulation in 9 day old Mock-treated (A) and 6-BAP treated L*er* plants for 6 hrs (B), 12 hrs (C), and 24 hrs (D). *pTCSn*::*mGFP5-ER* reporter expression in 9 day old Mock-treated (E) and 6-BAP treated L*er* plants for 6 hrs (F), 12 hrs (G), and 24 hrs (H). The SAMs showing the expression of *pWUS*::*dsRed-N7* transcriptional reporter which accumulates mostly in the apical L3 and Basal L3 layers upon Mock (I) and 6-BAP treatment for 24 hrs (J). A slight lateral expansion of WUS expression domain by 1–2 cell layers has been observed upon 6-BAP treatment for 24 hrs (J). The number of *WUS* positive cells in different SAM layers as measured by the expression of transcriptional reporter (*pWUS*::*dsRed-N7*) upon Mock and 6-BAP treatment for 24 hrs (K) (n = 12) is compared to the number of cells that accumulate WUS protein in different SAM layers as measured by the translational reporter (*pWUS*::*eGFP-WUS*) upon Mock and 6-BAP treatment for 24 hrs (n = 7) (L). Error bars for (K-L) represent standard error. The cell layers in SAMs are marked; the L1 and the L2 are monolayers. The multilayer L3 has been divided into the apical L3 layer and the basal L3 layers. The pith is located beneath the basal L3 layers. Insets for each image show the areas identified by black arrowheads at 4x zoom and white arrowheads show the boundaries of the reporter accumulation. Autofluorescence is denoted by grey arrowheads and is characterized by multiple foci in a single cell. eGFP and mGFP-ER (green) are overlaid on FM4-64 (red) plasma membrane stain in (A-H). The dsRed-N7 (red) is overlaid on DIC images (I-J). The scale bars = 50 μm for all images.

We tested the effect of exogenous application of 10 μM 6-benzylaminopurine (6-BAP), which is sufficient to activate transcription of a known cytokinin responsive gene, the type A *ARABIDOPSIS RESPONSE REGULATOR 5* (*ARR5*) [22], on the accumulation of WUS protein. Mock-treated *pWUS*::*eGFP-WUS* plants revealed *pWUS*::*eGFP-WUS* fluorescence primarily in the nuclei of the apical L3 and basal L3 cells of the SAM, and low levels of accumulation in the L1 and the L2 layers and also in the pith (Fig 1A). Upon 6 hrs of 6-BAP treatment, the *pWUS*::*eGFP-WUS* accumulation remained mostly nuclear and extended into the most basally located L3 cells and into the underlying pith (Fig 1B) (n = 8). By 12 hrs of 6-BAP treatment, *pWUS*::*eGFP-WUS* nuclear accumulation continued in the basal L3 layers and the pith, along with an increased accumulation in the L2 and the apical L3 layers (Fig 1C) (n = 5). By 24 hrs of 6-BAP treatment, the cells in the basal L3 layer and the pith also showed increased accumulation of the protein, with some accumulation appearing outside of the nuclei (Fig 1D and 1L) (n = 7). The cells located in L2 layer revealed a minimal increase in *pWUS*::*eGFP-WUS* accumulation even starting at 6 hrs, yet cells in the L1 layer did not show an increase in *pWUS*::*eGFP-WUS* accumulation until 24 hrs of 6-BAP treatment when a small increase was seen. The exposure of the *pWUS*::*dsRed-N7* transcriptional reporter to 10 μM 6-BAP did not reveal a dramatic change in the number of *WUS* expressing cells (Fig 1I–1K) (n = 7), which is consistent with an earlier study showing that cytokinin at concentrations within the physiological range did not induce *WUS* transcription [22]. Taken together, these observations reveal that exogenous application of cytokinin resulted in higher WUS protein accumulation without a detectable increase in *WUS* transcript levels.

An increase in the number of cells that accumulated higher WUS protein in the apical L3, the basal L3, and the pith cells when compared to the cells in the L1 and the L2 layers could be due to the increased cytokinin responsiveness of the deeper cell layers. To determine the relationship between cytokinin responsiveness and the WUS protein accumulation, we analyzed the spatial activation patterns of the synthetic cytokinin responsive promoter-*pTCSn*::*mGFP-ER* at various times after 10 μM 6-BAP treatment. The *pTCSn*::*mGFP-ER* expression in Mock-treated plants was restricted to the apical L3 and the basal L3 cells (Fig 1E) as also shown in an earlier study [25]. At 6 hrs of 6-BAP treatment a dramatic increase in *pTCSn*::*mGFP-ER* reporter activity in the basal L3, along with a slight increase in reporter activity was observed in the apical L3 layers, the underlying pith cells, and the vasculature tissue (Fig 1F) (n = 5). Continued 6-BAP treatment for 12 hrs (Fig 1G) (n = 4) and 24 hrs (Fig 1H) (n = 14) resulted in a dramatic increase in *pTCSn*::*mGFP-ER* reporter activity in all cells except in the centrally located L1 layer cells, while a few centrally located L2 layer cells activated the reporter weakly. In summary, a higher WUS protein accumulation was observed in cells that revealed a maximal response to cytokinin treatment.

### Cytokinin receptor mutants accumulate WUS at much lower levels in inner cell layers which also activate *CLV3* at higher levels

**Fig 2:**
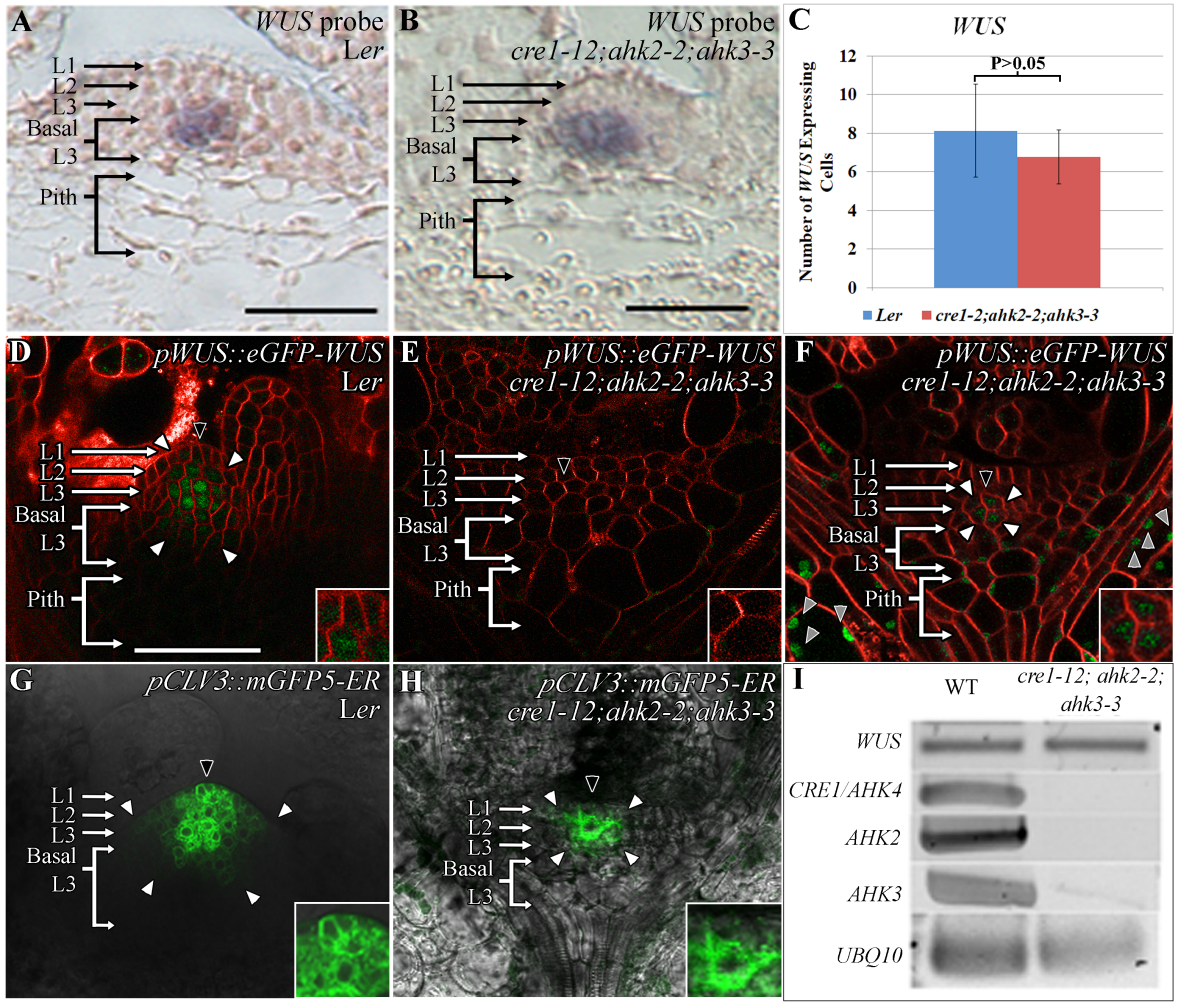
Lower WUS protein accumulation and altered *CLV3* expression in cytokinin receptor mutants. RNA *in situ* showing localization of *WUS* transcripts in L*er* (A) and in *cre1-12;ahk2-2*;*ahk3-3* (B) lines. The number of *WUS* expressing cells quantified from RNA *in situ* images in L*er* and *cre1-12;ahk2-2*;*ahk3-3*. P>0.05 as determined by a Student’s t-test (C). WUS protein (*pWUS*::*eGFP-WUS*) accumulation in L*er* (D) and in *cre1-12;ahk2-2*;*ahk3-3* (E-F). *cre1-12;ahk2-2*;*ahk3-3* plants showing no detectable *pWUS*::*eGFP-WUS* accumulation (E), a few *pWUS*::*eGFP-WUS* accumulating cells detected when imaged at 1.5x detector gain (F). The *pCLV3*::*mGFP5-ER* expression in L*er* (G) and *cre1-12;ahk2-2*;*ahk3-3* (H). Semi-quantitative RT-PCR of *WUS* and *CRE1*/*AHK4*, *AHK2*, and *AHK3* transcripts in wild type and *cre1-12;ahk2-2*;*ahk3-3* mutants (I). The cell layers in SAMs are marked; the L1 and the L2 are monolayers. The multilayer L3 has been divided into the apical L3 layer and the basal L3 layers. The pith is located beneath the basal L3 layers. Insets for each image show the areas identified by black arrowheads at 4x zoom and white arrowheads show the boundaries of reporter accumulation. Autofluorescence is denoted by grey arrowheads and is characterized by multiple foci in a single cell. eGFP (green) is overlaid on FM4-64 (red) plasma membrane stain in (D-F). mGFP5-ER is overlaid on DIC in (G-H). The scale bars = 50 μm for all images.

To test the requirement of the cytokinin signaling in WUS protein accumulation, we examined the spatial patterns of *WUS* transcription, *WUS* transcript levels, and WUS protein distribution in the cytokinin triple receptor mutant line, *cre1-12;ahk2-2*;*ahk3-3*, that has been shown to eliminate cytokinin signaling [28]. RNA *in situ* hybridization by using the antisense *WUS* probe revealed normal expression of *WUS* in deeper cell layers of both L*er* and the smaller sized *cre1-12;ahk2-2*;*ahk3-3* SAMs (Fig 2A and 2B) (n = 5). The number of *WUS* expressing cells were not significantly different in much smaller sized receptor mutant SAMs (Fig 2C). Moreover, the semi-quantitative RT-PCR analysis did not reveal a striking change in *WUS* transcript levels despite a dramatic downregulation of *CRE1*, *AHK2*, and *AHK3* transcript levels in *cre1-12;ahk2-2;ahk3-3* mutant line (Fig 2I). To study the effect of cytokinin signaling on WUS protein levels, we introduced the *pWUS*::*eGFP-WUS* described in an earlier study [20] into the *cre1-12;ahk2-2*;*ahk3-3* mutant line. In most plants, the *pWUS*::*eGFP-WUS* accumulation was undetectable (Fig 2E) (n = 18), except in images acquired at higher detector gain which revealed protein accumulation in 2–3 centrally located cells in the L2 and the apical L3 cell layers (Fig 2F) (n = 7). Taken together, this analysis reveals that cytokinin signaling is required for maintaining WUS protein levels.

In order to understand the significance of the decreased WUS accumulation in *cre1-12;ahk2-2*;*ahk3-3* mutants, we analyzed the expression pattern of *CLV3*, a direct transcriptional target of WUS [13]. The wild type *CLV3* expression is detected in a higher number of cells in the L1 layer when compared to the L2 and L3 layers (Fig 2G). The cytokinin receptor mutants revealed a much smaller *CLV3* expression domain with strongest expression detected in the two centrally located cells in the L2 layer (Fig 2H) (n = 6). A relatively higher *CLV3* expression in sub-epidermal cells could be due to the lower WUS detected in these cells showing the requirement of higher WUS to repress *CLV3* in inner layers. A relatively weaker *CLV3* expression in the L1 layer could be due to the limitation of WUS. These results are consistent with the WUS concentration-dependent regulation of *CLV3* and cytokinin as a spatial WUS stabilizing signal contributing to the spatial regulation of *CLV3*.

### Induction of cytokinin response in outer cell layers increases WUS protein accumulation

**Fig 3:**
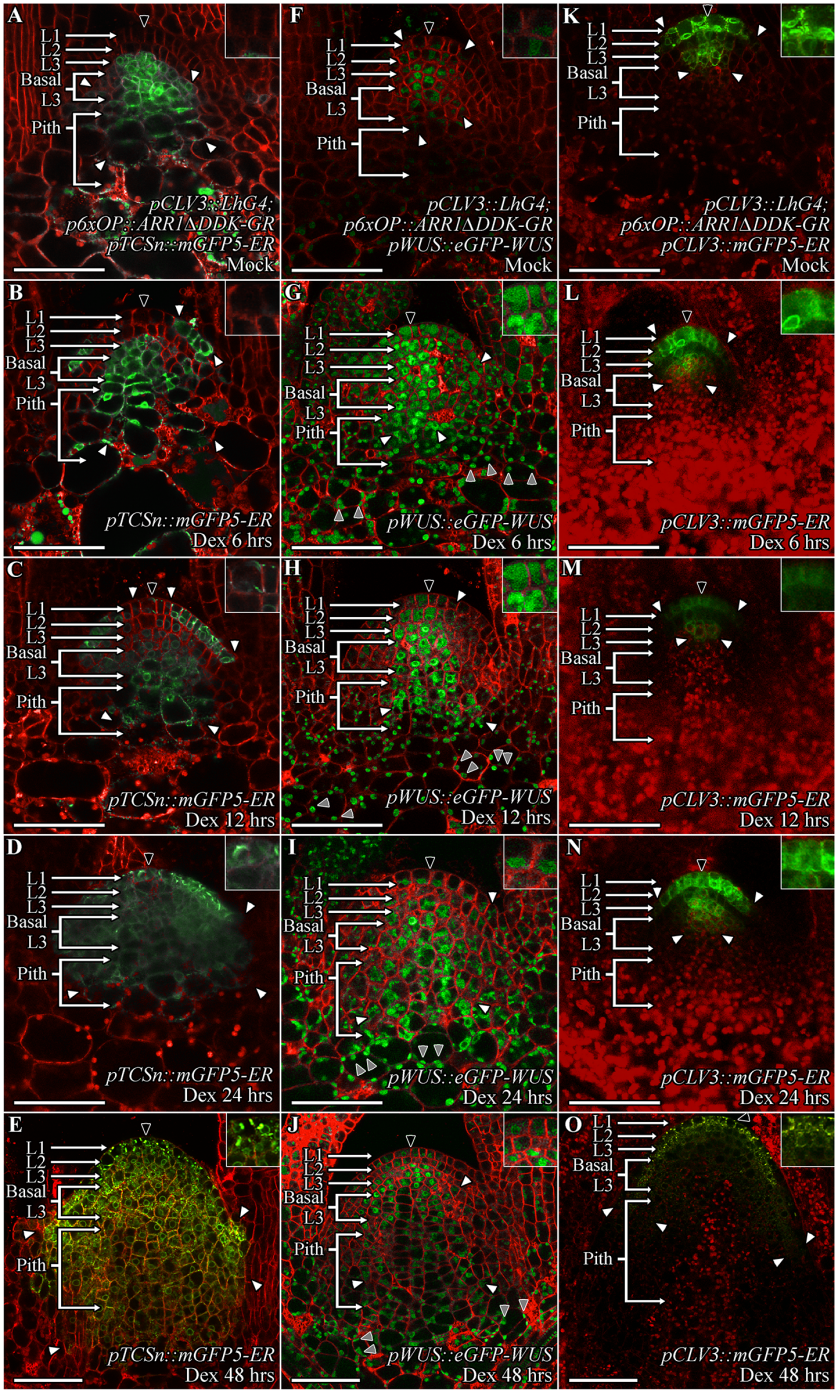
The ectopic activation of cytokinin signaling leads to higher WUS protein accumulation in SAMs, including the outer cell layers. The SAM images of nine day old *pCLV3*::*LhG4; p6xOP*::*ARR1ΔDDK-GR* seedling showing *pTCSn*::*mGFP5-ER* reporter expression (A-E), WUS protein (*pWUS*::*eGFP-WUS*) accumulation (F-J), and *CLV3* expression (*pCLV3*::*mGFP5-ER*) (K-O) in Mock-treated plants (A, F, K) and at 6 hrs (B, G, L), 12 hrs (C, H, M), 24 hrs (D, I, N), and 48hrs (E, J, O) after Dex treatment. The cell layers in SAMs are marked; the L1 and the L2 are monolayers. The multilayer L3 has been divided into the apical L3 layer and the basal L3 layers. The pith is located beneath the basal L3 layers. Insets for each image show the areas identified by black arrowheads at 4x zoom and white arrowheads show the boundaries of reporter accumulation. Autofluorescence is denoted by grey arrowheads and is characterized by multiple foci in a single cell. mGFP-ER and eGFP (green) are overlaid on FM4-64 (red) plasma membrane stain. The scale bars = 50 μm for all images.

The results presented in previous sections suggest that higher cytokinin response in the apical L3 and the basal L3 cells may promote WUS protein stability. The WUS protein that migrates into the overlying L1 and L2 cells, and into the underlying pith cells that all exhibit limited cytokinin response may become unstable. The exogenous application of cytokinin was unable to induce higher levels of cytokinin response in the L1 and the L2 layers, which correlates with lower levels of WUS protein accumulation (Fig 1A–1D). To test whether activation of the cytokinin signaling was sufficient to stabilize the protein in the L1 and the L2 layers, we ectopically expressed a type B ARABIDOPSIS RESPONSE REGULATOR 1 (ARR1), a TF which activates downstream targets upon phosphorylation by the cytokinin signaling pathway. The deletion of the phospho-transceiver domain (ΔDDK) has been shown to activate cytokinin signaling constitutively even in the absence of cytokinin, and the fusion of ARR1ΔDDK to the hormone binding domain of the rat glucocorticoid receptor (GR) has been shown to induce cytokinin response upon Dex treatment [29, 30]. We expressed *ARR1ΔDDK-GR* in the CZ by using the two-component system consisting of the *pCLV3*::*LhG4* driver together with *p6xOP*::*ARR1ΔDDK-GR*. To test the ability of this system in inducing ectopic cytokinin signaling, we monitored *pTCSn*::*mGFP5-ER* expression at 6 hrs, 12 hrs, 24 hrs, and 48 hrs after Dex treatment. The *pTCSn*::*mGFP5-ER* expression after 6 hrs of Dex treatment was elevated in the RM and a few cells in the PZ of the L1 layer (Fig 3B) (n = 6) compared to Mock-treated (Fig 3A) plants. By 12 hrs of Dex treatment, the L1 layer expression expanded inwards towards the CZ, and also into the basal L3 cells and the pith but at slightly reduced levels (Fig 3C) (n = 9). The L2 layer showed weakest response appeared to be the least sensitive to ARR1ΔDDK-GR induction along with the L1 cells in the CZ. At 24 hrs after Dex treatment, the *pTCSn*::*mGFP5-ER* expression was observed in all cell layers of SAMs, which was accompanied by an overall increase in SAM size (Fig 3D) (n = 5). The *pTCSn*::*mGFP5-ER* expression at 48 hrs after Dex treatment intensified further along with the increase in SAM size (Fig 3E) (n = 4). These observations show that expression of *ARR1ΔDDK-GR* in the CZ can induce cytokinin signaling. The spatial expression of *CLV3* promoter was restricted to the CZ across all time points after *ARR1ΔDDK-GR* induction (Fig 3K–3O) suggesting that the staggered centripetal pattern of induction of cytokinin response in the CZ and in the basal L3 layers is not due to the misexpression of the *CLV3* promoter but it is a non-cell autonomous effect.

Next, we monitored the fate of the *pWUS*::*eGFP-WUS* protein, and after 6 hrs of Dex treatment *pWUS*::*eGFP-WUS* levels increased in all layers of the SAM, including the L1 and the L2 layers (Fig 3G and S1A Fig) (n = 3). At 12 and 24 hrs after Dex treatment, *pWUS*::*eGFP-WUS* continued to accumulate at higher levels in the L2 and L3 layers, the basal L3 layers, and the pith, with a slight decrease in the L1 layer (Fig 3H and S1A Fig) (n = 6). Finally, at 48 hrs after Dex treatment, WUS protein was maintained at higher level in the L2 and the apical L3 layers while a decrease in protein level was observed in the L1 layer and in few cells in the pith (Fig 3J; S1A Fig) (n = 5). The analysis of *WUS* transcript pattern at 48 hrs of Dex treatment revealed an expanded *WUS* expression domain in deeper cell layers but the *WUS* transcripts were largely absent from the L1 and the L2 cell layers (S1B and S1C Fig) (n = 2). These results show that increase in WUS protein accumulation is not due to the *de novo WUS* transcription in the L1 and the L2 layers and also suggest that the ARR1-mediated activation of *WUS* transcription requires rib meristem context. The higher WUS accumulation in the L1 and the L2 layers could be due to the higher mobility of the protein from inner layers. However, higher WUS protein accumulation in the inner layers upon 6-BAP treatment was not sufficient for higher WUS accumulation in the L1 and the L2 layers. Taken together, these results show that ectopic induction of cytokinin response in the L1 and the L2 layers was sufficient to partially stabilize WUS.

### Cytokinin can offset destabilization of WUS observed upon ectopic overexpression

**Fig 4:**
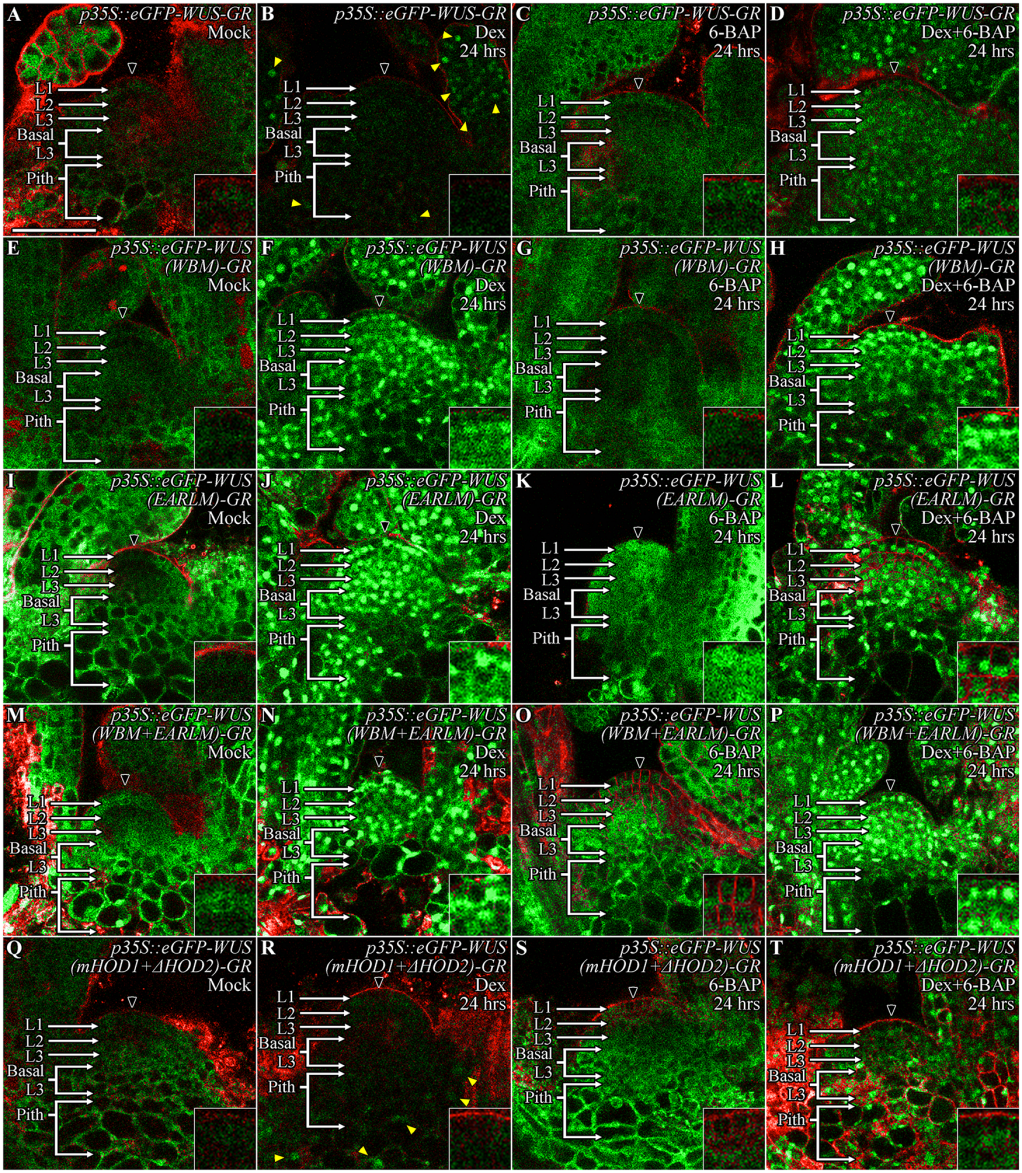
Cytokinin can offset ectopic WUS overexpression induced instability, which depends on the degron-like function of the WUS-box but does not require transcriptional activity of WUS. The SAMs showing the accumulation of ubiquitously expressed wild type (*p35S*::*eGFP-WUS-GR*), WUS-box mutant (*p35S*::*eGFP-WUS (WBM)-GR*), EAR-like domain mutant (*p35S*::*eGFP-WUS (EARLM)-GR*), double mutants of WBM mutant with the EARLM domain mutant (*p35S*::*eGFP-WUS (WBM+EARLM)-GR*), and double mutants of mHOD1 and ΔHOD2 (*p35*::*eGFP-WUS (mHOD1+ΔHOD2)-GR)* versions in Mock-treated (A, E, I, M, Q), Dex-treated for 24 hrs (B, F, J, N, R), 6-BAP-treated for 24 hrs (C, G, K, O, S), and combined Dex+6-BAP treated for 24 hrs (D, H, L, P, T) plants. 6-BAP treatment alone led to increase in cytoplasmically-localized WUS in wild type (C), EARLM (K), and the double mutants of mHOD1 and ΔHOD2 (S) while the WBM (G) and the double mutants of WBM and EARLM (O) did not reveal a striking increase in protein levels. Dex-treatment alone led to a dramatically lower levels of protein accumulation in cells located in the SAM (shown by the black arrowhead) in comparison to cells located in the developing leaves and the pith (yellow arrowheads) in wild type (B) and the double mutants of mHOD1 and ΔHOD2 (R) while the combined Dex and 6-BAP treatment improved protein accumulation both in wild type (D) and the double mutants of mHOD1 and ΔHOD2 (T). The Dex treatment of the WBM (F), EARLM (J) and the double mutants of WBM and EARLM (N) led to stable nuclear accumulation, though the WBM (F) did not show tight nuclear localization seen with the EARLM (J) and the WBM plus EARLM double mutants (N). The combined Dex and 6-BAP treatment (H, L, P) did not reveal a striking difference from that of the Dex alone treatment (F, J, N) in all three WUS forms. The cell layers in SAMs are marked; the L1 and the L2 are monolayers. The multilayer L3 has been divided into the apical L3 layer and the basal L3 layers. The pith is located beneath the basal L3 layers. Insets for each image show the areas identified by black arrowheads at 4x zoom. eGFP (green) is overlaid on FM4-64 (red) plasma membrane stain. The scale bars = 50 μm for all images.

Since the spatial accumulation of WUS involves several interconnected processes: nuclear-cytoplasmic partitioning, its intercellular movement, and stability, a steady state analysis alone is not sufficient to implicate cytokinin in the regulation of protein stability. Moreover, since cytokinin has been shown to increase *WUS* transcription [22], an unambiguous argument about cytokinin involvement in regulating WUS protein stability requires transient analysis by using *WUS* expressed from a heterologous promoter not regulated by cytokinin. Our earlier study has shown that the Dex inducible form of ubiquitously expressed-*p35S*::*eGFP-WUS-GR*, resulted in sequential destabilization, starting from the CZ within 6 hrs of Dex treatment and extending into the lateral edge of the PZ and the inner cell layers of the RM within 24 hrs of Dex treatment [20]. To test whether cytokinin can counter the instability, we co-treated wild type *p35S*::*eGFP-WUS-GR* seedlings with both 10 μM Dex and 10 μM 6-BAP. The 24 hrs treatment of *p35S*::*eGFP-WUS-GR* seedlings with 6-BAP alone resulted in an increase in fluorescence levels in the cytoplasm, especially in deeper cell layers (Fig 4C) (n = 5) when compared to the Mock-treated controls (Fig 4A) which is consistent with the higher activation of cytokinin response in these cells (Fig 1E–1H). The simultaneous treatment with both 10 μM Dex and 10 μM 6-BAP resulted in protein accumulation in the nuclei of all cells, including those that are located in the central part of the SAM (Fig 4D) (n = 11) when compared to the Dex treatment alone (Fig 4B) (n = 5). Though the protein accumulated at a relatively lower level in central part of the SAM when compared to the neighboring cells (Fig 4D). Taken together, these experiments show that cytokinin was able to prevent destabilization of WUS observed upon ectopic overexpression, revealing that cytokinin signaling likely acts directly on the WUS protein. Earlier studies have shown that higher levels of cytokinin can induce *WUS* transcription [21–24] gradually over several days [31]. It is possible that a higher threshold of cytokinin signaling and acquisition of cellular competence together induce *WUS* transcription. A relatively insensitive mechanism of cytokinin that induces *WUS* transcription could provide a long-term control of *WUS* expression, while the highly sensitive effect of stabilizing the WUS protein could enable rapid response over a shorter timescale.

### The transcriptional regulatory domains-acidic domain and WUS-box respond to cytokinin

**Fig 5:**
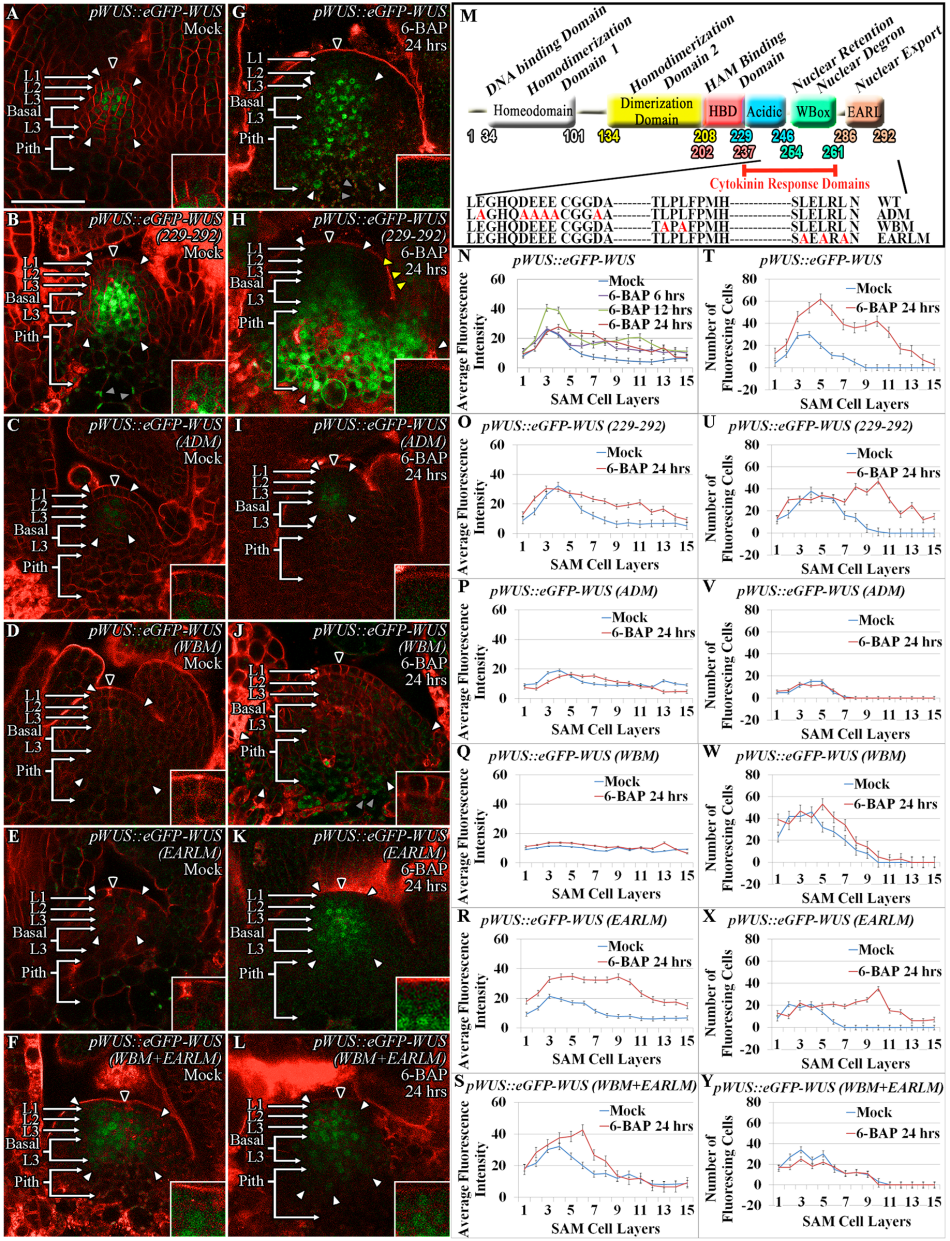
Cytokinin signaling acts on the transcriptional regulatory domains to stabilize WUS. Accumulation of various mutant forms of the WUS protein, expressed from the WUS promoter, 24 hrs after Mock (A-F) and 6-BAP treatment (G-L). *pWUS*::*eGFP-WUS* (A, G), *pWUS*::*eGFP-WUS (229–292)* (B, H), *pWUS*::*eGFP-WUS (ADM)* (C, I), *pWUS*::*eGFP-WUS (WBM)* (D, J), *pWUS*::*eGFP-WUS (EARLM)* (E, K) and *pWUS*::*eGFP-WUS (WBM+EARLM)* (F, L). A sketch showing the functions of WUS domains and associated amino acid changes in the mutant versions (M). Quantification of the relative fluorescence levels (N-S) and the number of fluorescing cells (T-Y) in Mock and 6-BAP treated plants. Error bars for (N-Y) represent standard error. The cell layers in SAMs are marked; the L1 and the L2 are monolayers. The multilayer L3 has been divided into the apical L3 layer and the basal L3 layers. The pith is located beneath the basal L3 layers. Insets for each image show the areas identified by black arrowheads at 4x zoom, white arrowheads show the boundaries of reporter accumulation, and yellow arrowheads point to WUS protein accumulation in developing leaves. Autofluorescence is denoted by grey arrowheads and is characterized by multiple foci in a single cell. eGFP (green) is overlaid on FM4-64 (red) plasma membrane stain. The scale bars = 50 μm for all images.

To further test whether cytokinin directly acts on the WUS protein, we utilized the series of mutant WUS proteins developed in an earlier study [20] (Fig 5M). The N-terminal DNA binding homeodomain also required for homodimerization (HOD1) together with the centrally located 74-aa stretch (amino acids 134–208) homodimerization domain (HOD2) restrict WUS protein diffusivity (Fig 5M) [20]. Additionally, the 63-aa stretch (amino acids 229–292) located at the C-terminus of the protein is sufficient for differential nuclear accumulation of WUS, as it influences nuclear-cytoplasmic partitioning and protein stability [20]. Therefore, we tested the responsiveness of the last 63 amino acid stretch of *pWUS*::*eGFP-WUS* (amino acids 229–292) (Fig 5B) to exogenous 6-BAP application. The 24 hrs of 6-BAP treatment led to a much broader accumulation of the truncated eGFP-WUS (amino acids 229–292) (Fig 5H, 5O and 5U) (n = 7), than the full length eGFP-WUS (Fig 5G, 5N and 5T) showing that this part of WUS contains signals for sensing cytokinin. The spread of the truncated eGFP-WUS (amino acids 229–292) further into the PZ and developing leaves is likely due to the increased mobility of this smaller and non-dimerizable form of the WUS protein [20].

To fine map the cytokinin sensing region, we tested the response of the mutant versions of the acidic domain, the WUS-box, and the EAR-like domain that were expressed from the WUS promoter [20]. A previous study has shown that deletion of the acidic domain destabilizes the protein as the mutant protein accumulated only in very few cells in the L3 layer, which could be due to the large-scale effects on the protein structure [20]. Therefore, we generated the *pWUS*::*eGFP-WUS (ADM)* construct by introducing point mutations in the acidic domain that previously have been shown to affect transcriptional activity [32]. The acidic domain mutant form of WUS accumulated at very low levels in very few cells (5–6 cells) located in the L3 layer (Fig 5C, 5P and 5V) (n = 5). Exogenous application of 6-BAP to the *pWUS*::*eGFP-WUS (ADM)* plants failed to induce the protein accumulation in the apical L3, the basal L3 and the pith cells, showing that the acidic domain is required for cytokinin sensing (Fig 5I, 5P and 5V) (n = 9). The WUS-box (*pWUS*::*eGFP-WUS (WBM)*) mutant version which has been shown to result in dramatic non-nuclear accumulation [20], also responded poorly to 6-BAP application and therefore revealed its essential function in cytokinin sensing (Fig 5D, 5J, 5Q and 5W) (n = 8). In contrast, the 6-BAP application was able to induce the accumulation of the EAR-like domain mutant version of WUS (*pWUS*::*eGFP-WUS (EARLM)*) in the basal L3 and the pith cells (Fig 5E, 5K, 5R and 5X) (n = 4). Variable accumulation of the *pWUS*::*eGFP-WUS (EARLM)* mutant version in different lines also suggests that these lines may have accumulated sub-threshold levels of WUS, leading to various degrees of instability (S2A–S2F Fig). The double mutant of the WUS-box and the EAR-like domain (*pWUS*::*eGFP-WUS (WBM+EARLM)*) accumulated stably in the nuclei of cells in the RM and the CZ (Fig 5F), but failed to respond robustly to 6-BAP application, again confirming the requirement of the WUS-box for sensing cytokinin (Fig 5L, 5S and 5Y) (n = 8). Collectively, these results show that the acidic domain and the WUS-box are essential for sensing cytokinin signaling.

### Degradation in cytokinin deficient region requires the WUSCHEL-box but not transcriptional activity

Our previous analysis showed that the last 63-aa stretch of WUS contains destabilizing signals [20]. The acidic domain functions as one of the cytokinin sensors along with the WUS-box. The EAR-like domain functions in nuclear export [20]. However, the EAR-like domain mutant lines showed various degrees of instability (S2A–S2F Fig) [20] when compared to the EAR-like domain and WUS-box double mutant which accumulated stably. The WUS-box mutant expressed from the *WUS* promoter accumulated uniformly in cells located both within and outside the RM, suggesting that the WUS-box may be required for destabilizing WUS in cells outside the RM [20]. However, it is also possible that non-nuclear accumulation could have improved stability. In addition, since the WUS-box is also required for transcriptional activity, the loss of transcriptional function might have led to improved stability. To address these issues, we carried out a transient analysis of the wild type and mutant versions of the WUS protein by using a Dex-inducible system.

We expressed the wild type, the WUS-box (WBM), the EAR-like domain (EARLM), and the double mutant (WBM+EARLM) forms of WUS as glucocorticoid receptor (GR) fusions from the ubiquitous (*p35S*) promoter. As shown in earlier study [20], the wild type-*p35S*::*eGFP-WUS-GR*, failed to accumulate in the CZ after 24 hrs of Dex treatment, however it did accumulate in the nuclei of the basal L3 cells, the pith, the PZ, and the leaves (Fig 4B) (n = 5). The EAR-like domain mutant *p35*::*eGFP-WUS (EARLM)-GR* (Fig 4I and 4J) (n = 16) accumulated in nuclei throughout the SAMs upon 24 hrs of Dex treatment in a manner consistent with its role in nuclear export. The WUS-box mutant *p35*::*eGFP-WUS (WBM)-GR* (Fig 4E and 4F) (n = 11), and the WBM and EARLM double mutant *p35*::*eGFP-WUS (WBM+EARLM)-GR* (Fig 4M and 4N) (n = 18) also accumulated uniformly in SAMs upon 24 hrs of Dex treatment. A relatively higher nuclear accumulation of Dex-treated WBM when expressed from the ubiquitous promoter (Fig 4F) as opposed to the non-nuclear accumulation observed when expressed from the WUS promoter [20] suggests that the nuclear export machinery may be limiting. Though these results show that both the EAR-like domain and the WUS-box are required for destabilization, the instability associated with the EAR-like mutants expressed from the WUS promoter suggests that it may not function directly in destabilization [20]. The stable accumulation of the WBM suggests either that it could function as a degron, or that the transcriptional activity provided by the WUS-box is necessary for destabilization.

**Fig 6:**
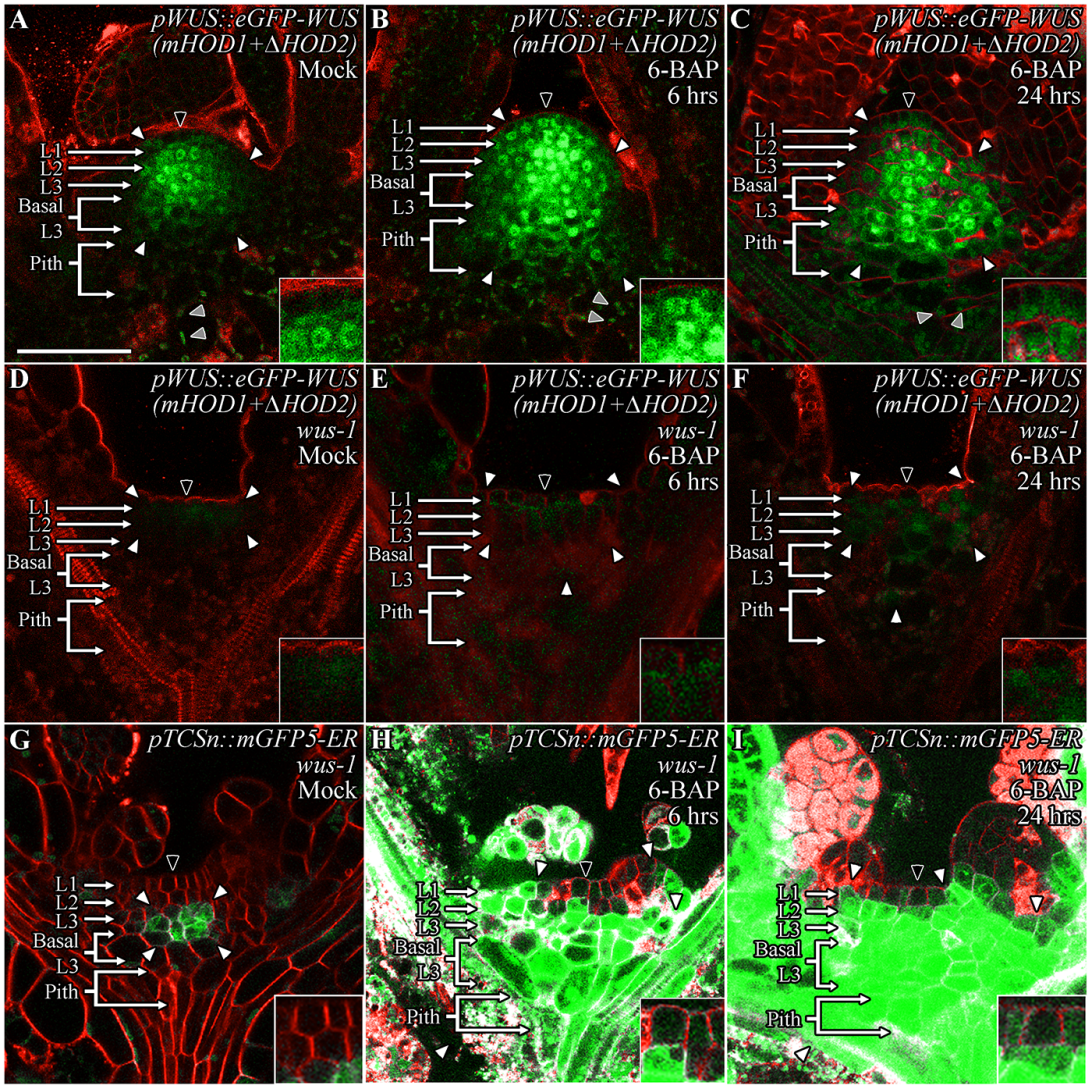
Transcriptional activity/functional WUS is required for stabilizing the WUS protein. The *pWUS*::*eGFP-WUS (mHOD1+ΔHOD2)* accumulation in wild type SAMs upon Mock (A), 6 hrs (B), and 24 hrs (C) of 6-BAP treatments. Note the diffuse accumulation of WUS and the response to 6-BAP treatments, particularly in the pith cells in (B-C). The *pWUS*::*eGFP-WUS (mHOD1+ΔHOD2)* accumulation in *wus-1* SAMs upon Mock (D), 6 hrs (E) and 24 hrs (F) of 6-BAP treatments. Note the poor accumulation of WUS in (D) and the much weaker response to 6-BAP treatments in (E-F). Cytokinin signaling response from the *pTCS*::*mGFP5-ER* in *wus-1* SAMs after Mock (G), 6 hrs (H), and 24 hrs (I) of 6-BAP treatments. The cytokinin response is clearly evident even after 6 hrs of treatment compared to Mock, demonstrating that cytokinin signaling is normal in *wus-1* SAMs and that the transcriptional activity/function of WUS is required for WUS stabilization. (A-F) are expressed from *pWUS*. The cell layers in SAMs are marked; the L1 and the L2 are monolayers. The multilayer L3 has been divided into the apical L3 layer and the basal L3 layers. The pith is located beneath the basal L3 layers. Insets for each image show the areas identified by black arrowheads at 4x zoom and white arrowheads show boundaries of the reporter accumulation. Autofluorescence is denoted by grey arrowheads and is characterized by multiple foci in a single cell. eGFP (green) is overlaid on FM4-64 (red) plasma membrane stain. The scale bar is 50 μm for all images.

To test whether transcriptional activity is required for destabilization, we generated an alternative transcriptionally dead version of WUS that leaves the wild type WUS-box intact. An earlier study has shown that the homodimerization deficient mHOD1 (single amino acid missense mutation G77E) and ΔHOD2 (deletion of amino acids 134–208) double mutant version fails to rescue *wus* null mutant phenotype (Fig 6D) [20]. The 24 hrs of Dex treatment of *p35*::*eGFP-WUS (mHOD1+ΔHOD2)-GR* (Fig 4Q and 4R) (n = 10) resulted in lower protein accumulation as seen with the wild type protein (Fig 4B), though the monomeric WUS accumulated at much lower levels especially in differentiating leaves (Fig 4R). Taken together, these results show that transcriptional activity/function of WUS is not required for its destabilization, therefore the stability observed with the WUS-box mutant version suggests that the wild type WUS-box could function as a degron in addition to its role in transcriptional activity. An earlier study has shown that the WUS-box mutant version of WUS accumulated stably when misexpressed in the L1 layer [13]. Our misexpression of the wild type WUS in the L1 layer (*pATML1*::*eGFP-WUS*) (n = 44) resulted in lack of visible protein accumulation, or in very few cases lower levels of non-nuclear accumulation (n = 6) (S4A–S4C Fig). This observation is consistent with the stable nuclear accumulation of the GR fusion form of WUS-box mutant version in outer cell layers supporting a role for the WUS-box in nuclear degradation in cells outside the RM that lack cytokinin signaling.

### Cytokinin can stabilize WUS irrespective of the sub-cellular distribution, DNA binding and oligomeric status

Cytokinin sensing through the WUS-box, which is also required for nuclear retention, might occur in the nucleus or through stabilization of WUS in the cytoplasm leading to higher nuclear import. To distinguish between these possibilities, we tested the responsiveness of the cytoplasmically-sequestered GR-fused forms of WUS to cytokinin. The 6-BAP application stabilized the mHOD1 and ΔHOD2 domain double mutant *p35*::*eGFP-WUS (mHOD1+ΔHOD2)-GR* (Fig 4S) (n = 11) and the EARLM *p35*::*eGFP-WUS (EARLM)-GR* (Fig 4K) in the cytoplasm similar to the wild type WUS *p35*::*eGFP-WUS-GR* (Fig 4C). The WBM *p35*::*eGFP-WUS (WBM)-GR* (Fig 4G) failed to respond as robustly as the EARLM *p35*::*eGFP-WUS (EARLM)-GR* (Fig 4K) which is consistent with the WUS-box being one of the cytokinin sensors (Fig 5J). The cytokinin induced increase of WUS in the cytoplasm was also observed with the last 63 amino acid stretch of the WUS (Fig 5H). Similar response patterns of the GR fused and the GR independent versions rules out the possibility the observed effects are artifacts of GR-mediated retention of the WUS in the cytoplasm. In addition, the use of heterologous *p35* promoter and the already translated GR-fused forms reveals that the cytokinin-induced WUS protein stability is a post-translational effect. These results show that the cytokinin can stabilize WUS protein in the cytoplasm irrespective of its oligomeric status or DNA binding, which could lead to a higher WUS pool available for nuclear import or diffusion into adjacent cells.

Next, we tested the combined effects of Dex and cytokinin treatments on the monomeric and other WUS mutant forms. The 24hr Dex treatment of the monomeric WUS variant *p35*::*eGFP-WUS (mHOD1+ΔHOD2)-GR* (Fig 4R) as shown in the previous section resulted in low protein accumulation similar to that of the wild type in the SAM, except in the pith and leaves where it accumulated at lower levels than the wild type protein (Fig 4B). The 24hr Dex plus 6-BAP treatments (Fig 4T) (n = 6) led to improved protein accumulation of the monomeric WUS, which was readily noticeable in inner cell layers, though the overall protein accumulation was lower and less nuclear than the wild type protein (Fig 4D). These results show that cytokinin can offset instability associated with the monomeric WUS, however, the DNA binding and homodimerization are required for higher nuclear accumulation. The combined 24hr treatments of 10 μM Dex and 10 μM 6-BAP did not result in a dramatic change in WUS forms that accumulated stably in the nucleus after Dex treatment alone, including *p35*::*eGFP-WUS (EARLM)-GR* (Fig 4L) (n = 18), *p35*::*eGFP-WUS (WBM)-GR* (Fig 4H) (n = 13), and *p35*::*eGFP-WUS (WBM+EARLM)-GR* (Fig 4P) (n = 3). These results suggest that the cytokinin signaling is largely responsible for stabilizing WUS until it reaches a level where it can independently remain stable.

### A functional WUS is required for stable WUS protein accumulation

Analyses presented in previous sections suggest that WUS can self stabilize at higher levels promoted either by cytokinin signaling or through higher nuclear import as shown in an earlier study [13]. To directly test whether transcriptional activity/function of WUS is required for self-stabilization, we introduced the transcriptionally dead-HOD1 and HOD2 double mutant form of WUS *pWUS*::*eGFP-WUS (mHOD1+ΔHOD2*) into *wus-1*, a null mutant of WUS. The mutant form of WUS was barely detectable in the *wus* mutant background (Fig 6D) when compared to the normal accumulation observed in the wild type background (Fig 6A). Earlier studies have shown that WUS promoter is active in *wus-1* mutant background [33] and the WUS promoter used in this study has been shown to rescue *wus* null mutant phenotype [11], which rules out lack of *WUS* transcription as the cause for lower WUS protein accumulation. The 6 hrs (Fig 6E) (n = 7) and 24 hrs (Fig 6F) (n = 7) treatments of 6-BAP were only able to minimally improve accumulation of the HOD1 and HOD2 double mutant form of WUS in the *wus-1* mutant background when compared to the 6 hrs (Fig 6B) (n = 3) and 24 hrs (Fig 6C) (n = 3) treatments of cytokinin in the wild type background, showing that functional WUS is required for higher protein accumulation and also for the cytokinin-mediated stabilizing effect. However, the inability of the HOD1 and HOD2 double mutant to respond maximally to cytokinin in *wus-1* mutant background could also be due to a decrease in cytokinin responsiveness of the *wus* mutants. To test this possibility, we analyzed the cytokinin response patterns in *wus-1* by using the cytokinin sensor *pTCSn*::*mGFP5-ER*. The *pTCSn* expression was observed in deeper cell layers of *wus-1* mutants (Fig 6G), a pattern that was comparable to that of the wild type (Fig 1E). The application of 6-BAP to *wus-1* mutants was able to fully induce the expression of *pTCSn* in the deeper cell layers, in the peripheral edges of the SAM, and in the developing leaves, yet it was excluded or expressed at extremely low levels in the L1 layer within 6 hrs (Fig 6H) (n = 5) of treatment. The overall expression intensified at 24 hrs (Fig 6I) (n = 7), similar to the response observed in the wild type SAM (Fig 1H) revealing that cytokinin response is maintained independently from WUS function. Together these results suggest that functional WUS is required for maintaining its own stability and that cytokinin may amplify this effect by enriching the protein in the nucleus.

## Discussion

WUS, a critical regulator of SAM development, is synthesized in the RM where it accumulates at higher levels and migrates into neighboring cells where it accumulates at lower levels [11]. WUS promotes stem cell maintenance by repressing differentiation factors to maintain differentiation program at a distance from the CZ [34]. Similarly WUS levels must also decrease in the basal L3 layers for their timely differentiation into the pith. Therefore, a precise control of the amount and spatial distribution of WUS protein accumulation is critical which could be regulated at the levels of synthesis, mobility and stability. The *WUS* has been shown to restrict its own transcription by activating the transcription of *CLV3*, which encodes a secreted peptide to activate receptor kinase pathway/s [10]. WUS has been shown to activate and repress *CLV3* transcription at lower and higher levels respectively to regulate *CLV3* levels and restrict its spatial expression to the CZ [13]. The RM-localized cytokinin signaling has been shown to activate *WUS* transcription [22, 31]. Our work reveals a highly sensitive role for localized cytokinin signaling, mediated by the well-characterized membrane-bound sensor histidine kinases, in stabilizing the WUS protein in the apical L3 cells to maintain spatial concentration which is required for spatial regulation of *CLV3* transcription and the meristem size. Moreover, the WUS-independent control of cytokinin response pattern, the stabilizer, may provide robustness to the WUS stability control and spatial regulation.

Our work shows that the higher instability of WUS outside the RM can be offset by cytokinin, which suggests that cytokinin may counter a ubiquitously present degradation signal. Our work also shows that WUS function/transcriptional activity is required for self-stabilization. Our previous work has shown that the WUS protein can be stabilized outside the RM by increasing the nuclear import [13] or by decreasing the nuclear export [20] suggesting that higher levels of nuclear WUS may negate the destabilizing signal. Perhaps at higher levels WUS could act as a better transcriptional repressor, as shown in the case of *CLV3* regulation [13], to downregulate genes in protein destabilization pathways, act at the post-translational level to block the destabilizing signal, or simply saturate the destabilization machinery. In an attempt to explore the regulation of WUS degradation, we treated *pWUS*::*eGFP-WUS* with proteasome inhibitor-MG132 treatment [35]. Within 4 hours of MG132 treatment, we observed a dramatic decrease (S5B Fig) (n = 8) or complete absence of eGFP-WUS signal (S5C Fig) (n = 12). This suggests that the nuclear degradation of WUS has more complex relationship with MG132 sensitive proteasome degradation pathway. It is possible that the degradation signal/factors necessary for WUS degradation are themselves sensitive to proteasomal degradation, and therefore the inhibition of proteasomal function would elevate their levels leading to a rapid WUS degradation. Future analysis aimed at identifying the degradation signal/s and the nature of the nuclear degradation pathway that destabilizes WUS may provide new insights.

**Fig 7:**
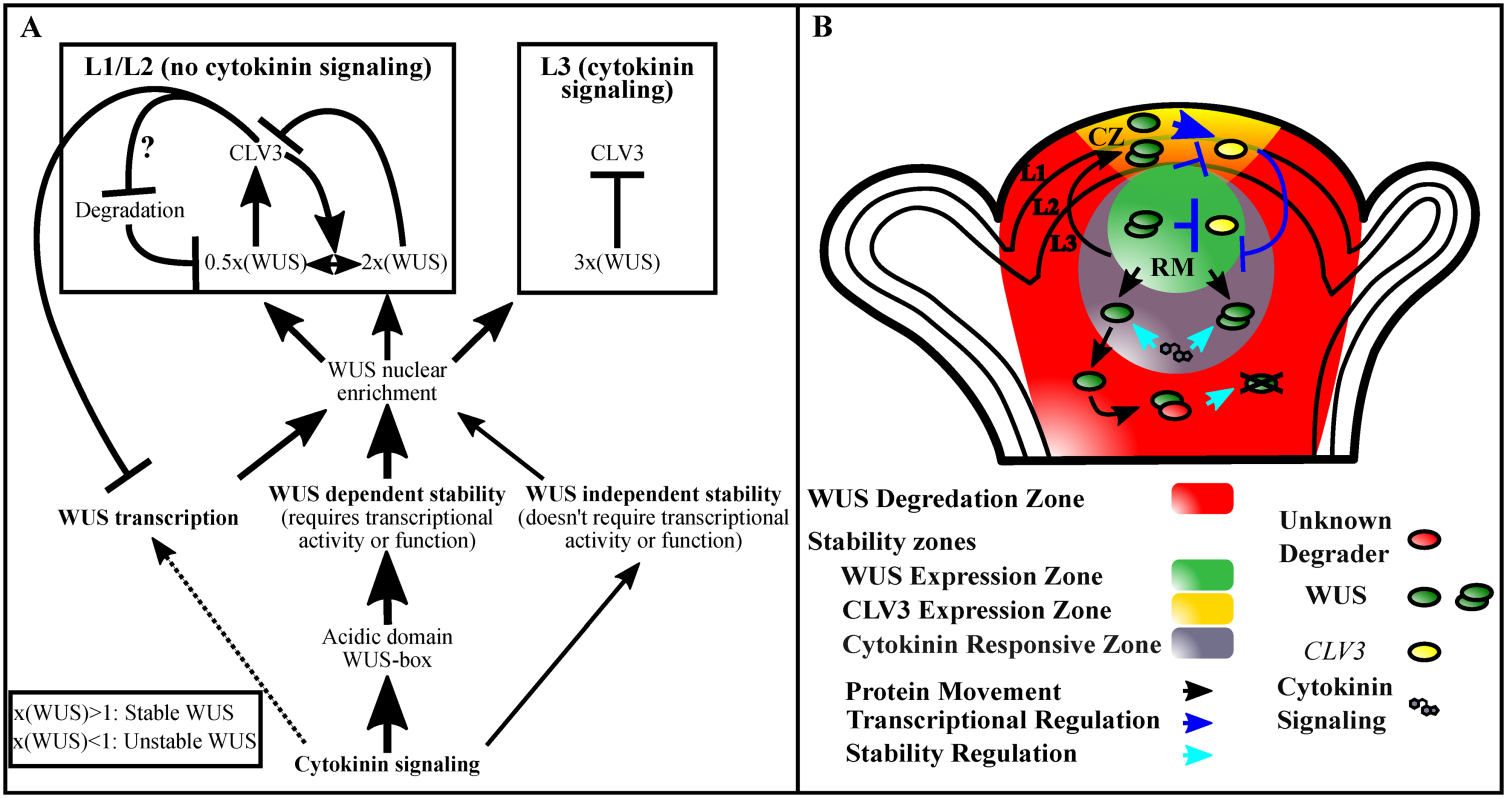
Summary of cytokinin effects on WUS and a model for the role of cytokinin in WUS-mediated regulation of the stem cell niche. (A) Cytokinin at higher level promotes *WUS* transcription. However, within the physiological range it stabilizes WUS protein by acting on the acidic domain and the WUS-box. The WUS-box is also required for nuclear retention and transcriptional regulation. A small amount of this stabilization can occur through a WUS-independent mechanism, while the majority of the cytokinin-mediated WUS stabilization requires WUS function. The consequent nuclear enrichment of WUS (3X) in the RM may mask degron activity of the WUS-box, leading to stability which in turn represses *CLV3* transcription. The L1 and the L2 layers of central part of the SAM are devoid of cytokinin signaling, where the degron activity of the WUS-box results in unstable WUS accumulation. However, CLV3 signaling partially offsets this instability in the L1 and the L2 layers. A relatively higher WUS (2X) represses *CLV3* which destabilizes WUS (0.5X), which in turn activates *CLV3*, thus forming a feedback loop at the post-translational level. The range of WUS levels represented (0.5X to 3X) are only relative. (B) A schematic showing the spatial representation of interactions that lead to stable and unstable WUS accumulation in space. Degradation system (red) is ubiquitously present. The cytokinin responsive zone (grey) in the RM stabilizes WUS protein. The wild type CLV3 signaling strength stabilizes WUS in the L1 and the L2 layers, while a lower CLV3 destabilizes WUS. Thus oscillating WUS levels mediate concentration-dependent activation and repression of *CLV3* transcription forming a self-sustaining feedback loop. *WUS* (green) and *CLV3* (Yellow) expression zones are marked.

In addition to cytokinin, the WUS-mediated activation of *CLV3* in the outer layers (the L1 and the L2) could offset WUS destabilization. This is because WUS fails to accumulate at higher level in the outer layers of *clv3* null mutants despite higher synthesis in the inner layers [13], even upon exogenous 6-BAP treatment (S3A and S3B Fig) (n = 4) which also fails to activate cytokinin response in outer layers (S3C and S3D Fig) (n = 14). Therefore, CLV3 could act as an additional signal to fine tune the WUS levels in the outer cell layers where cytokinin signaling is absent, allowing it to function as a concentration-dependent switch in regulating *CLV3* levels (Fig 7A). Future work will reveal whether CLV3-mediated signaling interferes with nuclear export leading to a higher nuclear WUS and stability or offsets nuclear destabilization directly.

Cytokinin signaling in the RM/L3 layers acts on the acidic domain and the WUS-box to stabilize WUS. The WUS-box is also required for nuclear retention [20]. The nuclear retention machinery acting in concert with the cytokinin signaling may lead to a higher nuclear accumulation, which could mask the degron like activity of the WUS-box leading to the stability of WUS. Outside the RM, the limitation of cytokinin signaling would lead to a reduced nuclear retention and an increased nuclear export through the activity of the EAR-like domain [20]. A net effect of these processes could lead to sub-threshold levels of WUS, which perhaps changes the protein conformation to expose the WUS-box to the destabilization signal. However, the causal relationship between nuclear export and degradation is not clear. The nuclear export could lead to WUS degradation in the cytoplasm as shown in the case of Aryl hydrocarbon receptor, a ligand (2,3,7,8- tetrachlorodibenzo-*p*-dioxin (TCDD)) activated nuclear TF [35], or nuclear export could simply decrease the nuclear levels of WUS leading to the nuclear degradation.

The WUS-box is also shown to be critical for transcriptional activity of WUS [32]. Earlier studies on TFs-Aryl hydrocarbon receptor [36], Transforming Growth Factor-ß activated SMAD2 [37], and Interferon-gamma activated STAT1 [38] have shown that the protein instability/higher turnover is coupled to transcriptional activation [39, 40]. Moreover, the unstable TFs in eukaryotes, and eubacteria [39] have been shown to use their transcriptional activation domains (TADs) as degrons. The differential utilization of the multifaceted WUS-box for nuclear enrichment in cytokinin rich cells and as a degron in neighboring cytokinin deficient cells provides a tighter spatial control of the regulation of local concentration of WUS and the transcriptional activation-repression switch. A recent study has suggested that WUS-box could function as a MAPkinase docking site required for generating phosphoisoforms of WUS [41]. Future work aimed at analyzing the *in vivo* relevance of phosphorylation may provide clues to multiple roles of the WUS-box in regulating WUS concentration and transcription.

## Materials and methods

### Plant growth, genotypes, and microscopy

*Arabidopsis* plant growth conditions were maintained as described in earlier studies [11]. For seedling imaging experiments, all plants were grown on ½ MS for 7–8 days and then transferred to plates or liquid ½ MS cultures containing either 10 μM 6-benzylaminopurine (6-BAP) (Acros Organics), 10 μM Dexamethasone (Dex) (Sigma), or 10 μM MG132 (Sigma) for the specified period. *clv3-2* [15], *wus-1* [8] and *cre1-12;ahk2-2;ahk3-3* [28] mutants have been described earlier. *pWUS*::*eGFP-WUS* [20], *pCLV3*::*LhG4* [14], *pTCS*::*mGFP5-ER* [25] and *pWUS*:*dsRed-N7*: [24], *pCLV3*::*mGFP5-ER* [19] have been described in earlier studies. *pTCS*::*mGFP5-ER* generated in Columbia ecotype was backcrossed twice to *clv3-2* and *wus-1*, and all observations were recorded in wild type *erecta* background. To examine the WUS protein localization in cytokinin receptor mutants, the *pWUS*::*eGFP-WUS* line was crossed with *cre1-12;ahk2-2;ahk3-3/+* plants. The F1 plants were backcrossed twice with *cre1-12;ahk2-2;ahk3-3/+* to generate *cre1-12;ahk2-2;ahk3-3/+* line that was homozygous for *pWUS*::*eGFP-WUS* in wild type *erecta* background.

To create the *p6xOP*::*ARR1ΔDDK-GR* vector, the DNA fragment of *ARR1ΔDDK-GR* was amplified using the primer pair MX318 and MX312rGR (see S1 Table) from the *p35S*::*ARR1ΔDDK-GR* plasmid (a kind gift from Dr. Takashi Aoyama, Institute for Chemical Research, Kyoto University), and cloned into the pENTR/D-TOPO vector. After confirming the sequence fidelity, the *ARR1ΔDDK-GR* fragment was inserted into *pMX6xOP*::*GW* (or *6xOP pzp222*) vector by the LR reaction and introduced into Landsberg *erecta* (L*er*) background. They were crossed to the *pCLV3*::*LhG4* driver line to generate homozygous plants. The PCR primers used for genotyping have been listed in S1 Table. The transgenic lines carrying the WUS mutant versions-the WUS-box (WBM), the EAR-like domain (EARLM) have been described in an earlier study [20]. The acidic domain mutant version is created by PCR mutagenesis by using the primers listed in S1 Table.

### RNA *in situ* hybridization

The seedlings of appropriate genotypes were grown for 7–8 days on ½ MS plates and transferred to Mock or 10 μM Dex plates for a specified period as necessary. Tissue collection, fixing, sectioning, and probe detection were performed was performed mostly as described earlier [19, 42, 43], with the following modifications: no salt was included in the ethanol dehydration series, and the RNase digestion step was not performed. Full length WUS cDNA was PCR amplified and cloned into pGEM T-easy (Promega) for probe synthesis using purified plasmids. RNA probe was generated with T7 RNA polymerase (Promega), labeled with dioxigenin-rUTP (Roche). Following hybridization, the probe was immuno-blotted with anti-DIG AB (Roche) and developed with Western Blue alkaline phosphatase (Promega).

### RT-PCR

For semi-quantitative RT-PCR experiments, three replicates each of seedling tissue from 9 plants were ground in liquid nitrogen. RNA was extracted according to the GeneJet Plant RNA Purification kit (Thermo Scientific). cDNA synthesis was performed using the ThermoScript RT-PCR System (Thermo Scientific). PCR fragments were amplified by employing 35 cycles (WUS), 35 cycles (CRE1), 35 cycles (AHK2), 30 and 35 cycles (AHK3) and 22 cycles (UBQ10). The primers used for amplification are listed in S1 Table.

### Tissue preparation and confocal microscopy

Seedlings were embedded in 3% agarose melted to 60°C to create a block for tissue sectioning. Excess agarose was trimmed off using a razor, tweezers, and a Zeiss Stemi 2000-C dissecting microscope. The embedded seedling was then oriented in a vertical position, and sliced into two halves using a feather razor blade. Each half was then immediately transferred to a 3% FM4-64x cell membrane staining solution for 10–15 minutes, and then placed on a slide with a coverslip in ample ddH_2_O. Plants were screened for optimal cut sections using a 20x objective on a Zeiss Axio Imager.A1 fluorescence microscope before final micrographs were captured with a 40x objective on a Leica SP5 Inverted Confocal microscope. eGFP fluorescence was detected with 488 nm excitation and an emission collection between 525 nm-550 nm. FM4-64x membrane stain and dsRed-N7 fluorescence were detected using 543 nm excitation with emission collected between 600 nm-650 nm and 575 nm-625 nm respectively.

### Quantification of fluorescence signal

Images from confocal microscopy were loaded into the Icy Bioimage software [44] (http://icy.bioimageanalysis.org) and isolated for the eGFP or dsRed-N7 channel. The HK Means and Active Contour plugins were used to detect cell counts. For fluorescence quantification analysis, ROI boxes were drawn in ImageJ [45] (https://imagej.nih.gov/ij/index.html) through the central column of SAM cells (30 μm wide and 100 μm tall) and plot profile was used to quantify fluorescence. This was performed on 4–8 confocal images per treatment, and values for each cell layer were averaged across all samples within a treatment. For cell count measurements, the number of fluorescing cells in each layer was counted for 4–8 confocal images per treatment and subsequently averaged across all samples.

## Supporting information

S1 Fig Quantification of WUS protein levels (*pWUS*::*eGFP-WUS*) and accumulation of *WUS* transcript pattern upon cytokinin induction in the central zone. *pWUS*::*eGFP-WUS* fluorescence levels in different cell layers of *pCLV3*::*LhG4; p6xOP*::*ARR1ΔDDK-GR* SAMs 6 hrs, 12 hrs, 24 hrs, and 48 hrs after Dex treatment is compared with Mock treated plants (A). RNA *in situ* showing localization of *WUS* in Mock (B) and Dex (C) treated *pCLV3*::*LhG4; p6xOP*::*ARR1ΔDDK-GR* plants. Error bars for (A) represent standard error. Scale bars are 50 μm for (B-C).
(TIF)

S2 Fig Variation of WUS accumulation in the eGFP-WUS (EARLM) expressing plants. The *pWUS*::*eGFP-WUS (EARLM)* construct ranged from low to high accumulation in smaller SAMs (A-C) to low to high accumulation across more cells in larger SAMs after 6-BAP treatment (D-F) across different lines. These variations could suggest a potential for WUS to stabilize in a larger area if it is permitted to reach a certain level. The image in (A) is the same used in Fig 5E. Insets for each image show the areas identified by black arrowheads at 4x magnification, white arrowheads show boundaries of the reporter accumulation, and yellow arrowheads point to WUS protein accumulation in developing leaves. eGFP (green) is overlaid on FM4-64 (red) plasma membrane stain. The scale bar is 50 μm for all images.
(TIF)

S3 Fig WUS accumulates at lower levels in outer cell layer despite higher synthesis in inner layers of cytokinin-treated *clv3-2* mutants. The *clv3-2* SAMs showing WUS protein (*pWUS*::*eGFP-WUS*) accumulation upon Mock (A) and 6-BAP 24 hrs (B) treatments. Note a relatively lower protein accumulation in the L1 and the L2 layers. The *clv3-2* SAMs showing *pTCSn*::*mGFP5-ER* cytokinin response upon Mock (C) and 6-BAP 24 hrs (D) treatments both show exclusion of the cytokinin response from the L1 and L2 layers, with 6-BAP treatment only increasing the levels of cytokinin response in the deeper L3 and pith cells. The L1 and the L2 are monolayers. The multilayer L3 has been divided into the apical L3 layer and the basal L3 layers. The pith is located beneath the basal L3 layers. Insets for each image show the areas identified by black arrowheads at 4x magnification and white arrowheads show boundaries of the reporter accumulation. eGFP and mGFP5-ER (green) are overlaid on FM4-64 (red) plasma membrane stain. The scale bars are 50 μm.
(TIF)

S4 Fig WUS accumulates very poorly when expressed directly in the L1 layer. eGFP-WUS expressed from the L1 layer (*pATML1*::*eGFP-WUS*) accumulates very poorly and mostly in the cytoplasm of the L1 layer (A, C) and L3 layer (B, C) of SAMs. Insets for each image show the areas identified by black arrowheads at 4x magnification. eGFP (green) is overlaid on FM4-64 (red) plasma membrane stain. The scale bar is 50 μm for all images.
(TIF)

S5 Fig WUS levels decrease when exposed to the proteasome inhibitor-MG132. *pWUS*::*eGFP-WUS* accumulation in wild type SAMs is highest in the L3 and deeper layers of the SAM and tapers off in the pith and the apical L1 and L2 layers (A). Treatment with MG132 results in reduced (B) and barely detectable (C) WUS accumulation. Insets for each image show the areas identified by black arrowheads at 4x magnification. eGFP (green) is overlaid on FM4-64 (red) plasma membrane stain. The scale bar is 50 μm for all images.
(TIF)

S1 Table Primers used in this study. (DOCX)

## References

[1] Rushlow CA, Han K, Manley JL, Levine M. The graded distribution of the dorsal morphogen is initiated by selective nuclear transport in Drosophila. Cell. 1989;59: 1165–1177. 2598265 10.1016/0092-8674(89)90772-1

[2] Nellen D, Burke R, Struhl G, Basler K. Direct and long-range action of a DPP morphogen gradient. Cell. 1996;85: 357–368. 8616891 10.1016/s0092-8674(00)81114-9

[3] Briscoe J, Ericson J. The specification of neuronal identity by graded Sonic Hedgehog signalling. Semin Cell Dev Biol. 1999;10: 353–362. doi: 10.1006/scdb.1999.0295 10441550 10.1006/scdb.1999.0295

[4] Steeves TA, Sussex IM. Patterns in plant development. Cambridge University Press; 1989.

[5] Reddy GV, Heisler MG, Ehrhardt DW, Meyerowitz EM. Real-time lineage analysis reveals oriented cell divisions associated with morphogenesis at the shoot apex of *Arabidopsis thaliana*. Development. 2004;131: 4225–4237. doi: 10.1242/dev.01261 15280208 10.1242/dev.01261

[6] Reddy GV. Live-imaging stem-cell homeostasis in the *Arabidopsis* shoot apex. Curr Opin Plant Biol. 2008;11: 88–93. doi: 10.1016/j.pbi.2007.10.012 18069047 10.1016/j.pbi.2007.10.012

[7] Barton MK. Twenty years on: the inner workings of the shoot apical meristem, a developmental dynamo. Dev Biol. 2010;341: 95–113. doi: 10.1016/j.ydbio.2009.11.029 19961843 10.1016/j.ydbio.2009.11.029

[8] Laux T, Mayer KF, Berger J, Jurgens G. The WUSCHEL gene is required for shoot and floral meristem integrity in *Arabidopsis*. Development. 1996;122: 87–96. 8565856 10.1242/dev.122.1.87

[9] Mayer KFX, Schoof H, Haecker A, Lenhard M, Jurgens G, Laux T. Role of WUSCHEL in Regulating Stem Cell Fate in the *Arabidopsis* Shoot Meristem. Cell. 1998;95: 805–815. 9865698 10.1016/s0092-8674(00)81703-1

[10] Schoof H, Lenhard M, Haecker A, Mayer KF, Jürgens G, Laux T. The stem cell population of Arabidopsis shoot meristems in maintained by a regulatory loop between the CLAVATA and WUSCHEL genes. Cell. 2000;100: 635–644. 10761929 10.1016/s0092-8674(00)80700-x

[11] Yadav RK, Perales M, Gruel J, Girke T, Jönsson H, Reddy GV. WUSCHEL protein movement mediates stem cell homeostasis in the *Arabidopsis* shoot apex. Genes Dev. 2011;25: 2025–2030. doi: 10.1101/gad.17258511 21979915 10.1101/gad.17258511 PMC3197201

[12] Daum G, Medzihradszky A, Suzaki T, Lohmann JU. A mechanistic framework for noncell autonomous stem cell induction in *Arabidopsis*. Proc of the Natl Acad of Sci. 2014;111:14619–14624. 10.1073/pnas.1406446111 PMC4210042 25246576

[13] Perales M, Rodriguez K, Snipes S, Yadav RK, Diaz-Mendoza M, Reddy GV. Threshold-dependent transcriptional discrimination underlies stem cell homeostasis. Proc of the Natl Acad of Sci. 2016;113: E6298–306. 10.1073/pnas.1607669113 PMC5068294 27671653

[14] Yadav RK, Tavakkoli M, Reddy GV. WUSCHEL mediates stem cell homeostasis by regulating stem cell number and patterns of cell division and differentiation of stem cell progenitors. Development. 2010;137:3581–3589. doi: 10.1242/dev.054973 20876644 10.1242/dev.054973

[15] Fletcher JC, Brand U, Running MP, Simon R, Meyerowitz EM. Signaling of cell fate decisions by CLAVATA3 in Arabidopsis shoot meristems. Science. 1999;283: 1911–1914. 10082464 10.1126/science.283.5409.1911

[16] Clark SE, Williams RW, Meyerowitz EM. The CLAVATA1 gene encodes a putative receptor kinase that controls shoot and floral meristem size in Arabidopsis. Cell. 1997;89: 575–585. 9160749 10.1016/s0092-8674(00)80239-1

[17] Brand U, Fletcher JC, Hobe M, Meyerowitz EM, Simon R. Dependence of Stem Cell Fate in *Arabidopsis* on a Feedback Loop Regulated by CLV3 Activity. Science. 2000;289: 617–619. 10915624 10.1126/science.289.5479.617

[18] Kondo T, Sawa S, Kinoshita A, Mizuno S, Kakimoto T, Fukuda H, et al A Plant Peptide Encoded by CLV3 Identified by in Situ MALDI-TOF MS Analysis. Science. 2006;313: 845–848. doi: 10.1126/science.1128439 16902141 10.1126/science.1128439

[19] Reddy GV, Meyerowitz EM. Stem-Cell Homeostasis and Growth Dynamics Can Be Uncoupled in the Arabidopsis Shoot Apex. Science. 2005;310: 663–667. doi: 10.1126/science.1116261 16210497 10.1126/science.1116261

[20] Rodriguez K, Perales M, Snipes S, Yadav RK, Diaz-Mendoza M, Reddy GV. DNA-dependent homodimerization, sub-cellular partitioning, and protein destabilization control WUSCHEL levels and spatial patterning. Proc of the Natl Acad of Sci. 2016;113: E6307–6315. 10.1073/pnas.1607673113 PMC5068338 27671631

[21] Lindsay DL, Sawhney VK, Bonham-Smith PC. Cytokinin-induced changes in CLAVATA1 and WUSCHEL expression temporally coincide with altered floral development in Arabidopsis. Plant Sci. 2006;170: 1111–1117.

[22] Gordon SP, Chickarmane VS, Ohno C, Meyerowitz EM. Multiple feedback loops through cytokinin signaling control stem cell number within the Arabidopsis shoot meristem. Proc of the Natl Acad of Sci. 2009;106: 16529–16534. 10.1073/pnas.0908122106 PMC2752578 19717465

[23] Meng WJ, Cheng ZJ, Sang YL, Zhang MM, Rong XF, Wang ZW, Tang YY, Zhang XS. Type-B ARABIDOPSIS RESPONSE REGULATORs Specify the Shoot Stem Cell Niche by Dual Regulation of WUSCHEL. Plant Cell. 2017;29: 1357–1372. doi: 10.1105/tpc.16.00640 28576846 10.1105/tpc.16.00640 PMC5502443

[24] Chickarmane VS, Gordon SP, Tarr PT, Heisler MG, Meyerowitz EM. Cytokinin signaling as a positional cue for patterning the apical–basal axis of the growing Arabidopsis shoot meristem. Proc of the Natl Acad of Sci. 2012;109: 4002–4007. 10.1073/pnas.1200636109 PMC3309735 22345559

[25] Zürcher E, Tavor-Deslex D, Lituiev D, Enkerli K, Tarr PT, Müller B. A robust and sensitive synthetic sensor to monitor the transcriptional output of the cytokinin signaling network in planta. Plant Physiol. 2013 3;161: 1066–1075. doi: 10.1104/pp.112.211763 23355633 10.1104/pp.112.211763 PMC3585579

[26] Gruel J, Landrein B, Tarr P, Schuster C, Refahi Y, Sampathkumar A, et al An epidermis-driven mechanism positions and scales stem cell niches in plants. Sci Adv. 2016;2: e1500989 doi: 10.1126/sciadv.1500989 27152324 10.1126/sciadv.1500989 PMC4846443

[27] Leibfried A, To JPC, Busch W, Stehling S, Kehle A, Demar M, et al WUSCHEL controls meristem function by direct regulation of cytokinin-inducible response regulators. Nature. 2005;438: 1172–1175. doi: 10.1038/nature04270 16372013 10.1038/nature04270

[28] Higuchi M, Pischke MS, Mähönen AP, Miyawaki K, Hashimoto Y, Seki M, et al In planta functions of the Arabidopsis cytokinin receptor family. Proc Natl Acad Sci. 2004;101: 8821–8826. doi: 10.1073/pnas.0402887101 15166290 10.1073/pnas.0402887101 PMC423279

[29] Sakai H, Aoyama T, Oka A. Arabidopsis ARR1 and ARR2 response regulators operate as transcriptional activators. Plant J. 2000;24: 703–711. 11135105 10.1046/j.1365-313x.2000.00909.x

[30] Sakai H, Honma T, Aoyama T, Sato S, Kato T, Tabata S, et al ARR1, a transcription factor for genes immediately responsive to cytokinins. Science. 2001;294: 1519–1521. doi: 10.1126/science.1065201 11691951 10.1126/science.1065201

[31] Wang J, Tian C, Zhang C, Shi B, Cao X, Zhang TQ, Zhao Z, Wang JW, Jiao Y. Cytokinin Signaling Activates WUSCHEL Expression during Axillary Meristem Initiation. Plant Cell. 2017;29(6): 1373–1387. doi: 10.1105/tpc.16.00579 28576845 10.1105/tpc.16.00579 PMC5502442

[32] Ikeda M, Mitsuda N, Ohme-Takagi M. Arabidopsis WUSCHEL Is a Bifunctional Transcription Factor That Acts as a Repressor in Stem Cell Regulation and as an Activator in Floral Patterning. Plant Cell. 2009;21: 3493–3505. doi: 10.1105/tpc.109.069997 19897670 10.1105/tpc.109.069997 PMC2798335

[33] Fujita H, Toyokura K, Okada K, Kawaguchi M. Reaction-diffusion pattern in shoot apical meristem of plants. PLoS One. 2011 3 29;6(3):e18243 doi: 10.1371/journal.pone.0018243 21479227 10.1371/journal.pone.0018243 PMC3066213

[34] Yadav RK, Perales M, Gruel J, Ohno C, Heisler M, Girke T, et al Plant stem cell maintenance involves direct transcriptional repression of differentiation program. Mol Syst Biol. 2013;9: 654 doi: 10.1038/msb.2013.8 23549482 10.1038/msb.2013.8 PMC3658276

[35] Davarinos NA, Pollenz RS. Aryl hydrocarbon receptor imported into the nucleus following ligand binding is rapidly degraded via the cytosplasmic proteasome following nuclear export. J Biol Chem. 1999:274: 28708–28715. 10497241 10.1074/jbc.274.40.28708

[36] Ma Q, Baldwin KT. 2, 3, 7, 8-Tetrachlorodibenzo-p-dioxin-induced Degradation of Aryl Hydrocarbon Receptor (AhR) by the Ubiquitin-Proteasome Pathway. Role of the transcription activation and DNA binding of AhR. J Biol Chem. 2000;275: 8432–8438. 10722677 10.1074/jbc.275.12.8432

[37] Lo RS, Massagué J. Ubiquitin-dependent degradation of TGF-beta-activated smad2. Nat Cell Biol. 1999;1: 472–478. doi: 10.1038/70258 10587642 10.1038/70258

[38] Kim TK, Maniatis T. Regulation of interferon-g-activated STAT1 by the ubiquitin-proteasome pathway. Science. 1996;273: 1717–1718. 8781235 10.1126/science.273.5282.1717

[39] Muratani M, Tansey WP. How the ubiquitin-proteasome system controls transcription. Nat Rev Mol Cell Biol. 2003;4: 192–201. doi: 10.1038/nrm1049 12612638 10.1038/nrm1049

[40] Liu H, Stone SL. Cytoplasmic degradation of the Arabidopsis transcription factor abscisic acid insensitive 5 is mediated by the RING-type E3 ligase KEEP ON GOING. J Biol Chem. 2013;288(28):20267–79. doi: 10.1074/jbc.M113.465369 23720747 10.1074/jbc.M113.465369 PMC3711294

[41] Dory M, Doleschall Z, Nagy SK, Ambrus H, Mészáros T, Barnabás B, Dóczi R. Kinase-Associated Phosphoisoform Assay: a novel candidate-based method to detect specific kinase-substrate phosphorylation interactions in vivo. BMC Plant Biol. 2016 9 21;16(1):204 doi: 10.1186/s12870-016-0894-1 27655033 10.1186/s12870-016-0894-1 PMC5031308

[42] Yadav RK, Girke T, Pasala S, Xie M, Reddy GV. Gene expression map of the *Arabidopsis* shoot apical meristem stem cell niche. Proc of the Natl Acad of Sci. 2009;106: 4941–4946. 10.1073/pnas.0900843106 PMC2660727 19258454

[43] Jackson D. In situ hybridization in plants Molecular plant pathology: a practical approach. Oxford University Press, Oxford 1991; 163–174.

[44] de Chaumont F. et al Icy: an open bioimage informatics platform for extended reproducible research. Nature Methods. 2012;9:690–696. doi: 10.1038/nmeth.2075 22743774 10.1038/nmeth.2075

[45] Schneider CA, Rasband WS, Eliceiri KW. NIH Image to ImageJ: 25 years of image analysis. Nature Methods. 2012;9:671–675. 22930834 10.1038/nmeth.2089 PMC5554542

