# Supplementary figures and images for: Cytokinin stabilizes WUSCHEL by acting on the protein domains required for nuclear enrichment and transcription
Source: PLoS Genet. 2018 Apr 16;14(4):e1007351. doi: 10.1371/journal.pgen.1007351 (PMC5919686; doi:10.1371/journal.pgen.1007351)

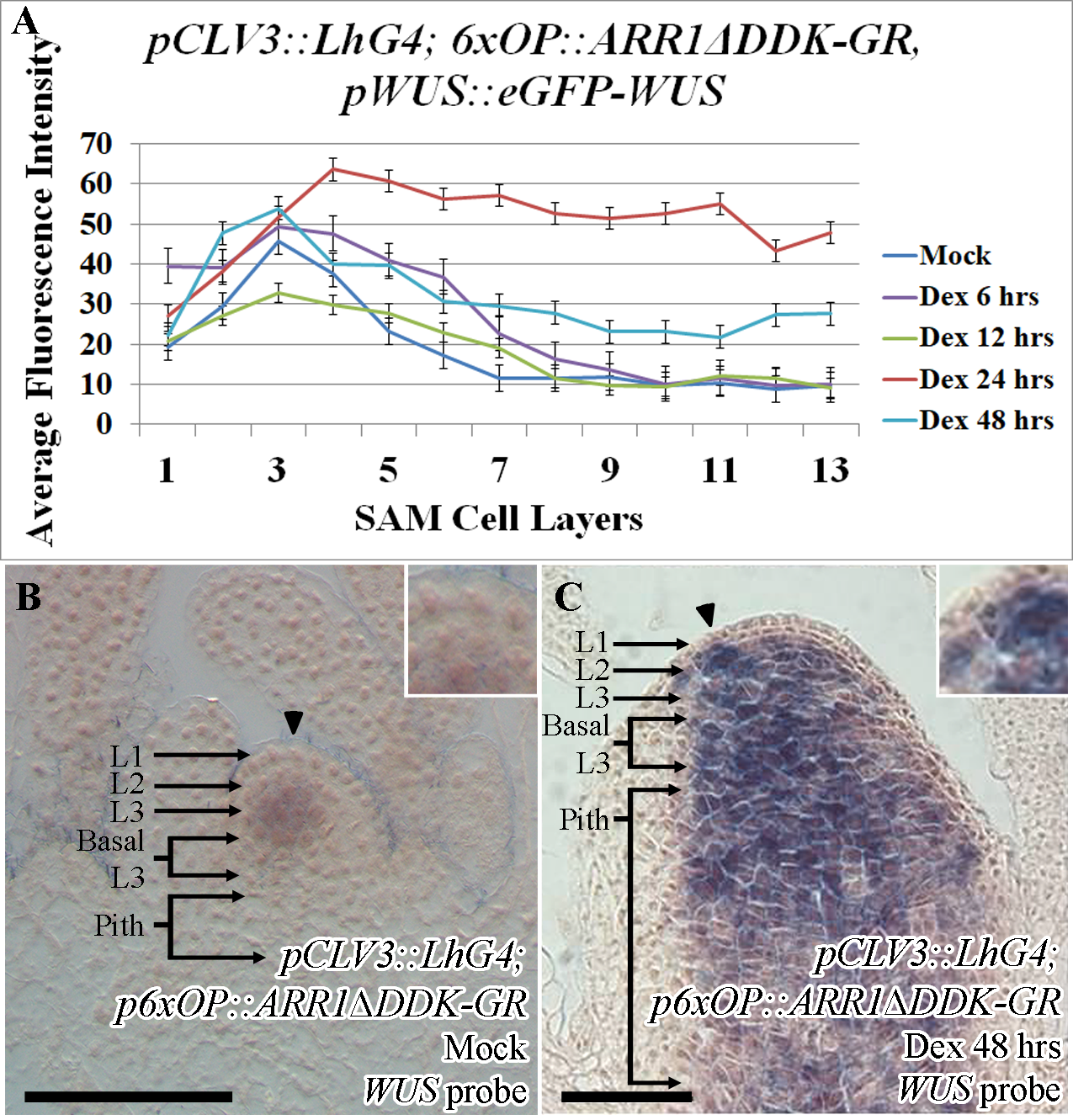

Supplement: S1 Fig — pWUS::eGFP-WUS fluorescence levels in different cell layers of pCLV3::LhG4; p6xOP::ARR1ΔDDK-GR SAMs 6 hrs, 12 hrs, 24 hrs, and 48 hrs after Dex treatment is compared with Mock treated plants (A). RNA in situ showing localization of WUS in Mock (B) and Dex (C) treated pCLV3::LhG4; p6xOP::ARR1ΔDDK-GR plants. Error bars for (A) represent standard error. Scale bars are 50 μm for (B-C). (TIF) [file pgen.1007351.s001.tif]

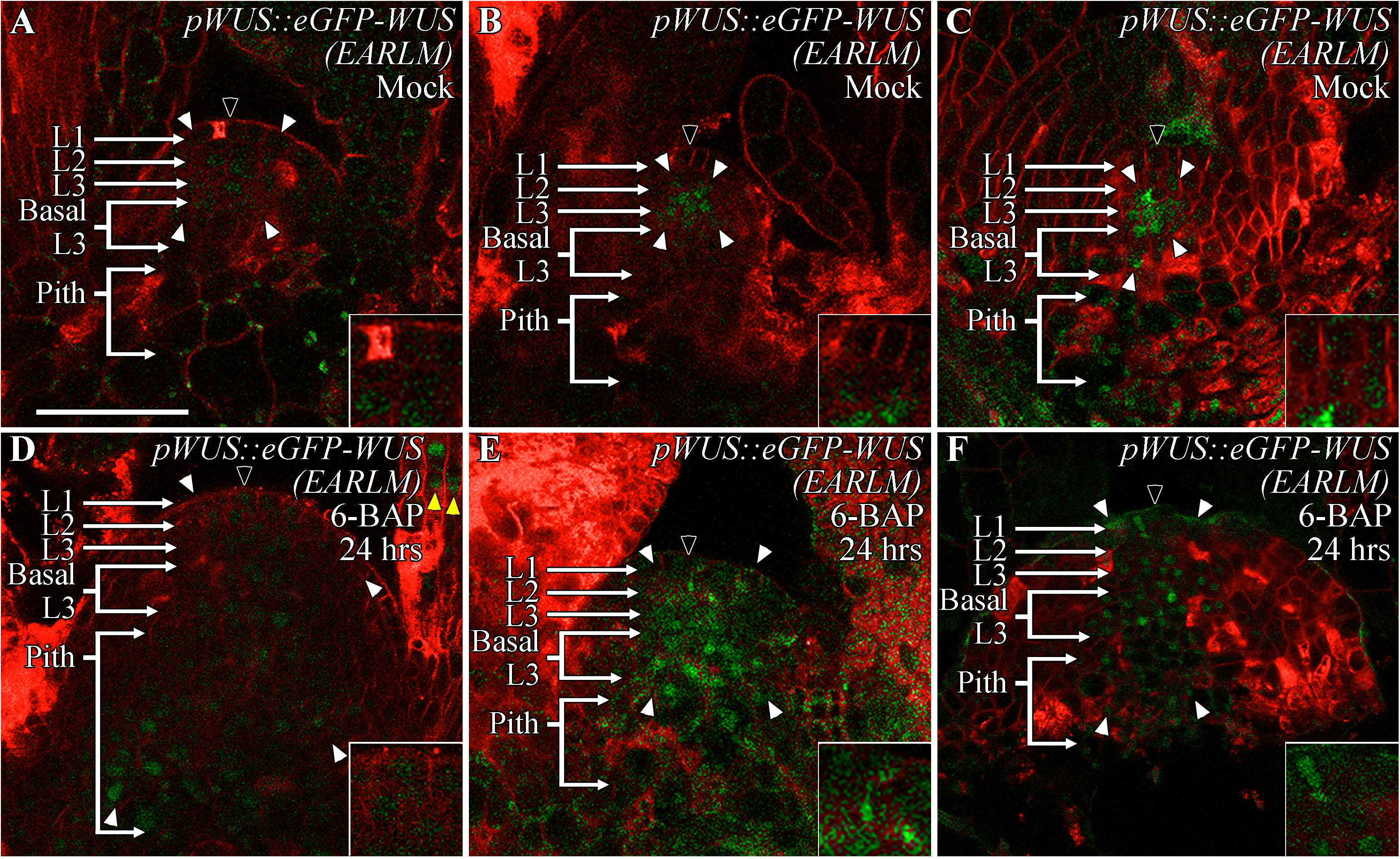

Supplement: S2 Fig — The pWUS::eGFP-WUS (EARLM) construct ranged from low to high accumulation in smaller SAMs (A-C) to low to high accumulation across more cells in larger SAMs after 6-BAP treatment (D-F) across different lines. These variations could suggest a potential for WUS to stabilize in a larger area if it is permitted to reach a certain level. The image in (A) is the same used in Fig 5E. Insets for each image show the areas identified by black arrowheads at 4x magnification, white arrowheads show boundaries of the reporter accumulation, and yellow arrowheads point to WUS protein accumulation in developing leaves. eGFP (green) is overlaid on FM4-64 (red) plasma membrane stain. The scale bar is 50 μm for all images. (TIF) [file pgen.1007351.s002.tif]

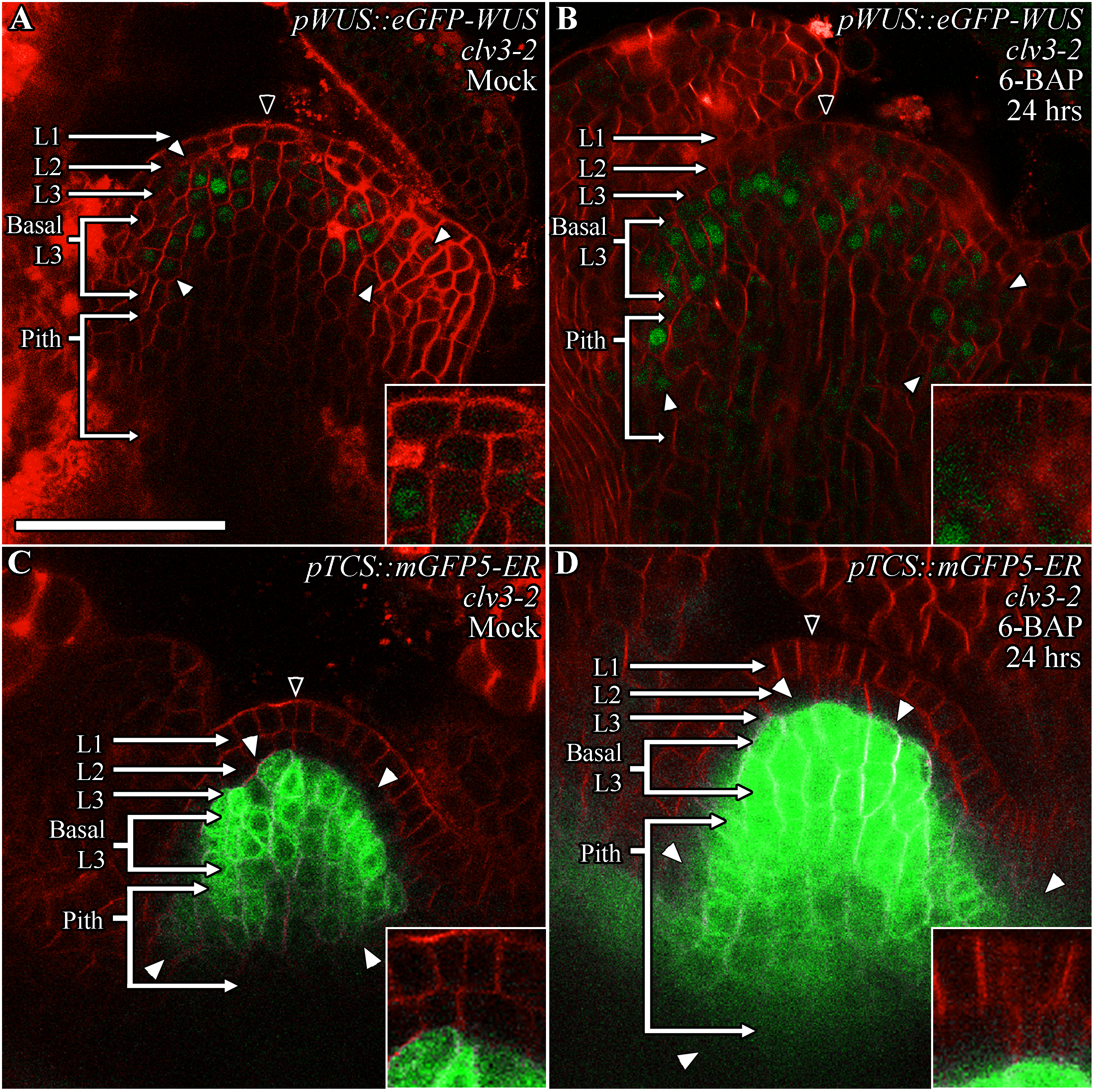

Supplement: S3 Fig — The clv3-2 SAMs showing WUS protein (pWUS::eGFP-WUS) accumulation upon Mock (A) and 6-BAP 24 hrs (B) treatments. Note a relatively lower protein accumulation in the L1 and the L2 layers. The clv3-2 SAMs showing pTCSn::mGFP5-ER cytokinin response upon Mock (C) and 6-BAP 24 hrs (D) treatments both show exclusion of the cytokinin response from the L1 and L2 layers, with 6-BAP treatment only increasing the levels of cytokinin response in the deeper L3 and pith cells. The L1 and the L2 are monolayers. The multilayer L3 has been divided into the apical L3 layer and the basal L3 layers. The pith is located beneath the basal L3 layers. Insets for each image show the areas identified by black arrowheads at 4x magnification and white arrowheads show boundaries of the reporter accumulation. eGFP and mGFP5-ER (green) are overlaid on FM4-64 (red) plasma membrane stain. The scale bars are 50 μm. (TIF) [file pgen.1007351.s003.tif]

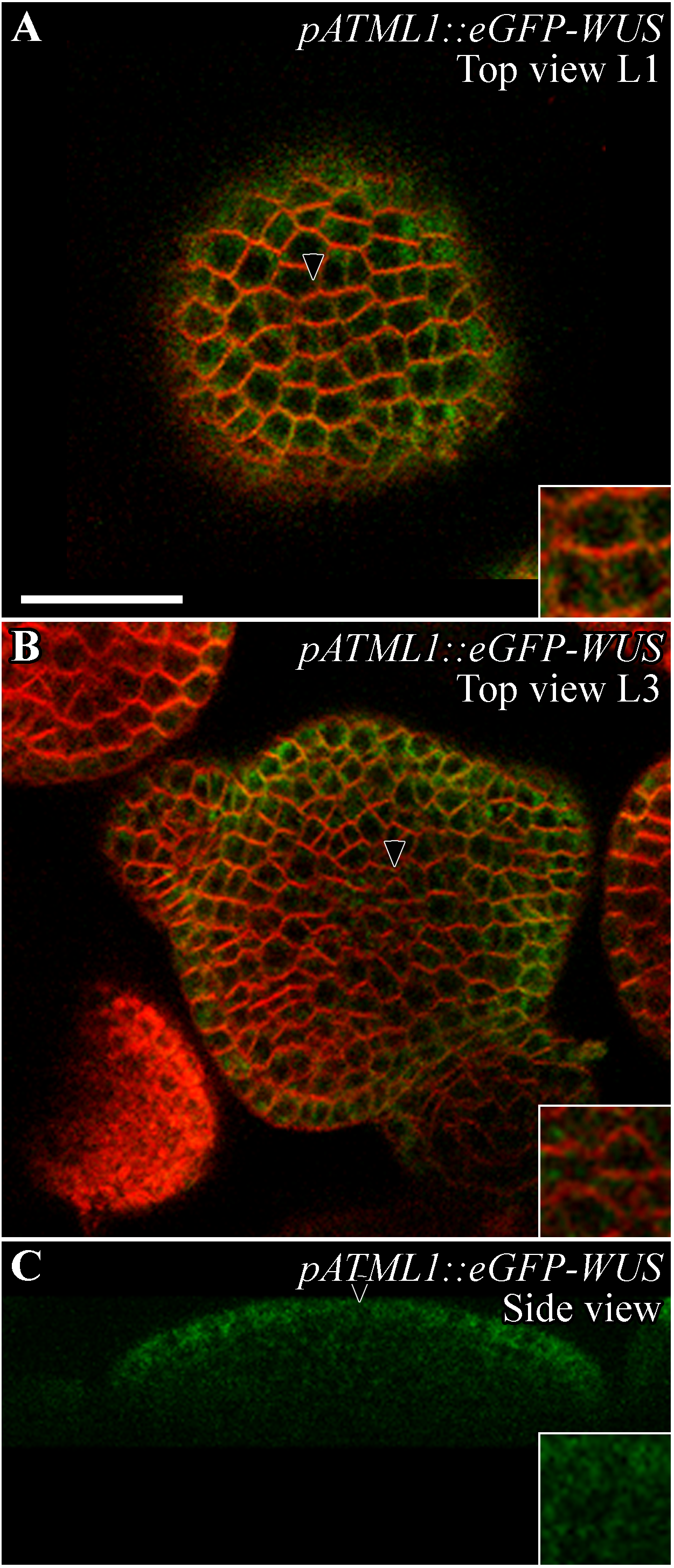

Supplement: S4 Fig — eGFP-WUS expressed from the L1 layer (pATML1::eGFP-WUS) accumulates very poorly and mostly in the cytoplasm of the L1 layer (A, C) and L3 layer (B, C) of SAMs. Insets for each image show the areas identified by black arrowheads at 4x magnification. eGFP (green) is overlaid on FM4-64 (red) plasma membrane stain. The scale bar is 50 μm for all images. (TIF) [file pgen.1007351.s004.tif]

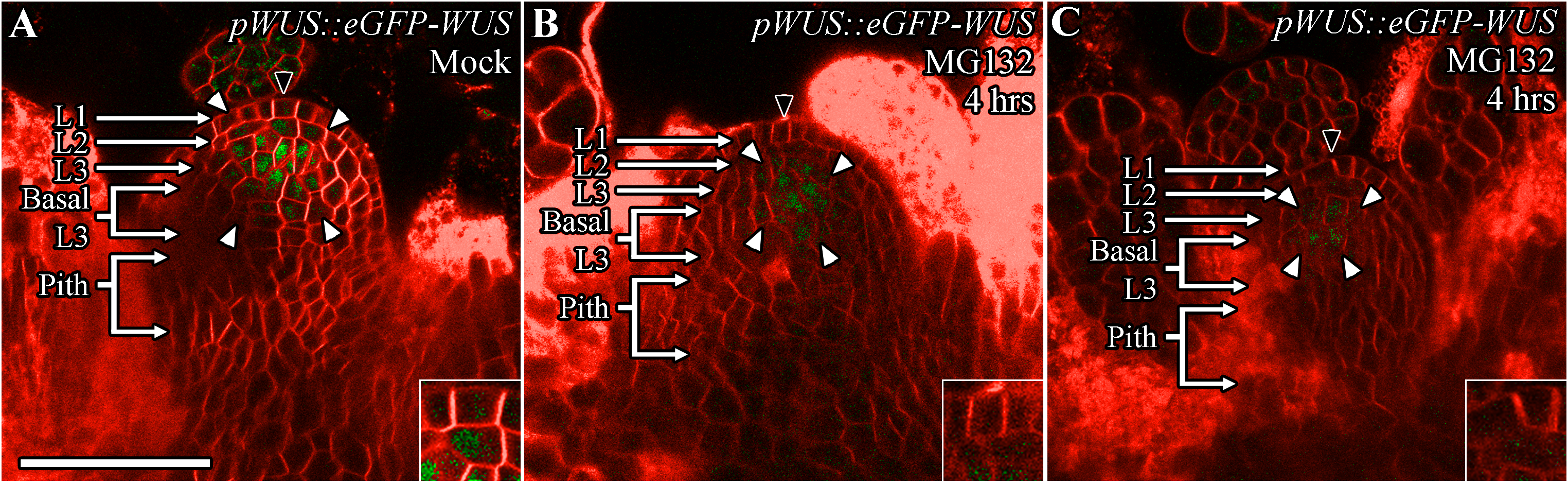

Supplement: S5 Fig — pWUS::eGFP-WUS accumulation in wild type SAMs is highest in the L3 and deeper layers of the SAM and tapers off in the pith and the apical L1 and L2 layers (A). Treatment with MG132 results in reduced (B) and barely detectable (C) WUS accumulation. Insets for each image show the areas identified by black arrowheads at 4x magnification. eGFP (green) is overlaid on FM4-64 (red) plasma membrane stain. The scale bar is 50 μm for all images. (TIF) [file pgen.1007351.s005.tif]
