# Supplementary material for: Cytokinin stabilizes WUSCHEL by acting on the protein domains required for nuclear enrichment and transcription
Source: PLoS Genet. 2018 Apr 16;14(4):e1007351. doi: 10.1371/journal.pgen.1007351 (PMC5919686; doi:10.1371/journal.pgen.1007351)
Supplement: S1 Table — (DOCX) [file pgen.1007351.s006.docx]

**Table S1. Primers used in this study**

| Gene | Primer | Sequence |
| --- | --- | --- |
| WUS | WUSRTF | ATCATGCAAGCTCAGGTACTGAATGT |
|  | WUSRTR | GAGCTTTAATCCCGAGCGACACCGG |
| UBIQUITIN10 | ubq10 5' | TTCACTTGGTCCTGCGTCTTCGTGTGGTTTC |
|  | ubq10 3' | CGAAGCGATGATAAAGAAGAAGTTCGACTTG |
| AHK2 | ATHK2-2F | GTCTATAACTTGTGAGCTCTTGAATC |
|  | ATHK2-2R | GCTCGTGTCATAGACAGCAAAGGTC |
| AHK3 | ATHK3-3F | CTTGTGATTGCGTTACTTGTTGCAC |
|  | ATHK3-3R | GCAGGCCTATGGTCCACAACCACAG |
| CRE1/AHK4 | CRE1-12F | GGAGAGCCTTCACCGGTTAGGG |
|  | CRE1-12R | AAGCTCTTGCATTTCATGGAAATC |
| LB primer | LB1 | GCCTTTTCAGAAATGGATAAATAGCCTTGCTTCC |
| ARR1ΔDDK-GR | MX318 | TTAGGAAGAGGAGAAGTGAATGGAGTGTAC |
|  | MX312rGR | TCATTTTTGATGAAACAGAAG |
| WUS acidic domain Mutation | 5' Fwd Acidic domainM | GCATGTGGTGGCGCTGCTTATCTGGAA |
|  | 5'Rev Acidic domainM | TGCTGCGGCTTGATGACCTGCTAGACCAAAC |
